# Distinct functions of FASCICLIN-LIKE ARABINOGALACTAN PROTEINS relate to domain structure

**DOI:** 10.1093/plphys/kiad097

**Published:** 2023-02-17

**Authors:** Yingxuan Ma, Thomas Shafee, Asha M Mudiyanselage, Julian Ratcliffe, Colleen P MacMillan, Shawn D Mansfield, Antony Bacic, Kim L Johnson

**Affiliations:** School of BioSciences, University of Melbourne, Parkville, VIC 3052, Australia; La Trobe Institute for Sustainable Agriculture and Food, Department of Animal, Plant and Soil Science, La Trobe University, Bundoora, VIC 3086, Australia; La Trobe Institute for Sustainable Agriculture and Food, Department of Animal, Plant and Soil Science, La Trobe University, Bundoora, VIC 3086, Australia; La Trobe Institute for Sustainable Agriculture and Food, Department of Animal, Plant and Soil Science, La Trobe University, Bundoora, VIC 3086, Australia; La Trobe Institute for Sustainable Agriculture and Food, Department of Animal, Plant and Soil Science, La Trobe University, Bundoora, VIC 3086, Australia; CSIRO, Agriculture and Food, CSIRO Black Mountain Science and Innovation Park, Canberra, ACT 2601, Australia; Department of Wood Science, University of British Columbia, Vancouver, BC V6T 1Z4, Canada; La Trobe Institute for Sustainable Agriculture and Food, Department of Animal, Plant and Soil Science, La Trobe University, Bundoora, VIC 3086, Australia; Sino-Australia Plant Cell Wall Research Centre, College of Forestry and Biotechnology, Zhejiang Agriculture and Forestry University, Lin’an, Hangzhou 311300, China; La Trobe Institute for Sustainable Agriculture and Food, Department of Animal, Plant and Soil Science, La Trobe University, Bundoora, VIC 3086, Australia; Sino-Australia Plant Cell Wall Research Centre, College of Forestry and Biotechnology, Zhejiang Agriculture and Forestry University, Lin’an, Hangzhou 311300, China

## Abstract

The role of glycoproteins as key cell surface molecules during development and stress is well established; yet, the relationship between their structural features and functional mechanisms is poorly defined. FASCICLIN-LIKE ARABINOGALACTAN PROTEINs (FLAs), which impact plant growth and development, are an excellent example of a glycoprotein family with a complex multidomain structure. FLAs combine globular fasciclin-like (FAS1) domains with regions that are intrinsically disordered and contain glycomotifs for directing the addition of *O*-linked arabinogalactan (AG) glycans. Additional posttranslational modifications on FLAs include *N*-linked glycans in the FAS1 domains, a cleaved signal peptide at the N terminus, and often a glycosylphosphatidylinositol (GPI) anchor signal sequence at the C terminus. The roles of glycosylation, the GPI anchor, and FAS1 domain functions in the polysaccharide-rich extracellular matrix of plants remain unclear, as do the relationships between them. In this study, we examined sequence–structure–function relationships of Arabidopsis (*Arabidopsis thaliana*) FLA11, demonstrated to have roles in secondary cell wall (SCW) development, by introducing domain mutations and functional specialization through domain swaps with FLA3 and FLA12. We identified FAS1 domains as essential for FLA function, differentiating FLA11/FLA12, with roles in SCW development, from FLA3, specific to flowers and involved in pollen development. The GPI anchor and AG glycosylation co-regulate the cell surface location and release of FLAs into cell walls. The AG glycomotif sequence closest to the GPI anchor (AG2) is a major feature differentiating FLA11 from FLA12. The results of our study show that the multidomain structure of different FLAs influences their subcellular location and biological functions during plant development.

## Teaser

Differences in subcellular location and biological functions of fasciclin-like arabinogalactan proteins rely on variations in their complex multidomain glycoprotein structure.

## Introduction

The properties of the polysaccharide-rich extracellular matrix (cell wall) define cellular morphology, tissue, and plant architecture and are essential for growth. The composition of walls varies depending on the plant species, cell type, tissue, developmental stage, and history of responses to abiotic and biotic stresses, each factor with the potential to modify the type and distribution of constituent polysaccharides within individual cells. Crucial to the wall's role as a cell surface sensor are proteins and glycoproteins that transduce and respond to external and internal cues (Ellis et al. 2010).

A major group of plant cell wall glycoproteins are the ARABINOGALACTAN PROTEINS (AGPs) that include proline-rich regions with glycomotifs that direct the addition of *O*-linked AG glycans (Johnson et al. 2017a, 2017b). Classical AGPs and AG peptides can be highly glycosylated (∼90% w/w), whereas chimeric AGPs, which include a recognized protein family (PFAM) domain, have been shown to be moderately glycosylated (Showalter et al. 2010; Johnson et al. 2017a, 2017b; Ma et al. 2018). AGPs have been implicated in regulating many aspects of plant growth and development including cell division and differentiation in embryo and postembryo development, male–female recognition in reproduction organs, seed mucilage cell wall development, stem secondary cell wall (SCW) development, root hair development, and root salt tolerance (Ellis et al. 2010). Despite their importance, the molecular mechanism of AGP function(s) has been enigmatic for decades. The AG glycans of AtAGP57C have been shown to covalently crosslink to pectin and arabinoxylan in primary cell walls, thereby contributing to structural integrity (Tan et al. 2012). Recently, a function for the glucuronic acid (GlcA) residues on the AG glycan of AGPs being involved in calcium complexing and signaling has been demonstrated based on the investigation of AG glycan synthesis mutants showing growth and development phenotypes (Lamport and Varnai 2013; Lopez-Hernandez et al. 2020). The FASCICLIN-LIKE ARABINOGALACTAN PROTEINS (FLAs), subclass of AGPs, are moderately glycosylated and contain other known signaling motifs (FAS1 domains) and might, therefore, be expected to additionally signal through protein–protein and/or protein–polysaccharide interactions. Many FLAs are additionally posttranslationally modified by attachment of a glycosylphosphatidylinositol (GPI) anchor that directs secretion to the outer leaflet of the plasma membrane (PM) and association with distinct lipid microdomains to co-locate signaling complexes (Johnson et al. 2003; Ellis et al. 2010).

FAS1 domains are ancient structures that have been identified in proteins from a wide range of taxa, including bacteria, insects, mammals, and plants (Bastiani et al. 1987; Elkins et al. 1990; Kim et al. 2000; Clout et al. 2003; Johnson et al. 2003; Takayama et al. 2006). Human periostin, transforming growth factor-*β*-induced protein IG-H3 (*β*IG-H3), and stabilin-1 contain tandem repeat FAS1 domains are the most extensively studied mammalian FAS1 proteins (Kii and Ito 2017). Periostin has been shown to directly interact with structural proteins, including fibronectin, tenascin, collagen, and *β*IG-H3, acting as a scaffold for extracellular organization and signaling (Bonnet et al. 2016; Ma et al. 2020). In plants, FAS1 domains almost exclusively occur in FLAs and are proposed to play parallel functions in the extracellular matrix. A large number of domain architectures occur in FLAs, with 18 distinct FAS1 types that predominantly occur either once or twice in a sequence in combination with a diverse array of AG glycomotif types (Johnson et al. 2003; Shafee et al. 2020). Typically, the FAS1 type is highly predictive (45%) of the associated AG profile. However, experimental information on the relationship between FAS1 and AG/GPI anchor signal sequence regions and the importance of specificity of biological function(s) are unclear. In addition, the functional specialization of the different FAS1 types remains unexplored.

The majority of FLAs with known biological roles have been characterized by Arabidopsis (*Arabidopsis thaliana*). The contribution of different FLA domains to biological function has only been investigated for FLA4, involved in root salt tolerance. Domain deletion studies showed that only 1 of the 2 FAS1 domains in FLA4, the C-terminal (Type A) FAS1, was necessary for its biological functions in response to salt stress (Xue et al. 2017). Deletion of the AG region in FLA4 was shown to influence secretion to the PM/wall, however, was still able to function during salt stress (Xue et al. 2017). A recent bioinformatics study was able to distinguish potential functional differences in FLA members that had previously been assigned to the same group (Shafee et al. 2020). For example, Group A included FLAs with a single FAS1 domain that can now be distinguished into FAS1 types L (FLA7), N (FLA6, FLA9, and FLA13), and O (FLA11 and FLA12) and distinct disordered region types. For example, FLA11 and FLA12 both function during SCW development in Arabidopsis and fall into different types that may indicate functional specificity.

FLA11 and FLA12 are involved in regulating the mechanical properties of SCWs in stems, but these studies have not revealed the underlying mechanism(s) of action at a molecular level (MacMillan et al. 2010). Our previous work identified early SCW initiation and changes in lignification in FLA11 overexpression (OE-FLA11) plants (Ma et al. 2022). These phenotypes, combined with our bioinformatics study of FLA sequence features, form the basis for investigating FLA domain structures and functional mechanisms. In this study, we generated plants with OE-FLA11 domain mutation/deletion variants for all predicted functional domains and investigated SCWs phenotypes and FLA subcellular location. FAS1, AG2, and GPI anchor domain specificity was then compared among FLA11, FLA12, and a pollen-specific FLA3 using domain swaps. Our data support a role for both the FAS1 and the glycomotif domains as signaling modules of FLAs that can control the process of differentiation during plant development and complement the recent findings that GlcAs on the glycans of AGPs signal through modulating calcium concentrations in the extracellular matrix (Lamport and Varnai 2013; Lopez-Hernandez et al. 2020). These findings also highlight the remarkable parallels between the functional roles of mammalian extracellular matrix proteoglycans and those of plants.

## Results

### Characterization of FLA11 functional domains

**Figure 1.**
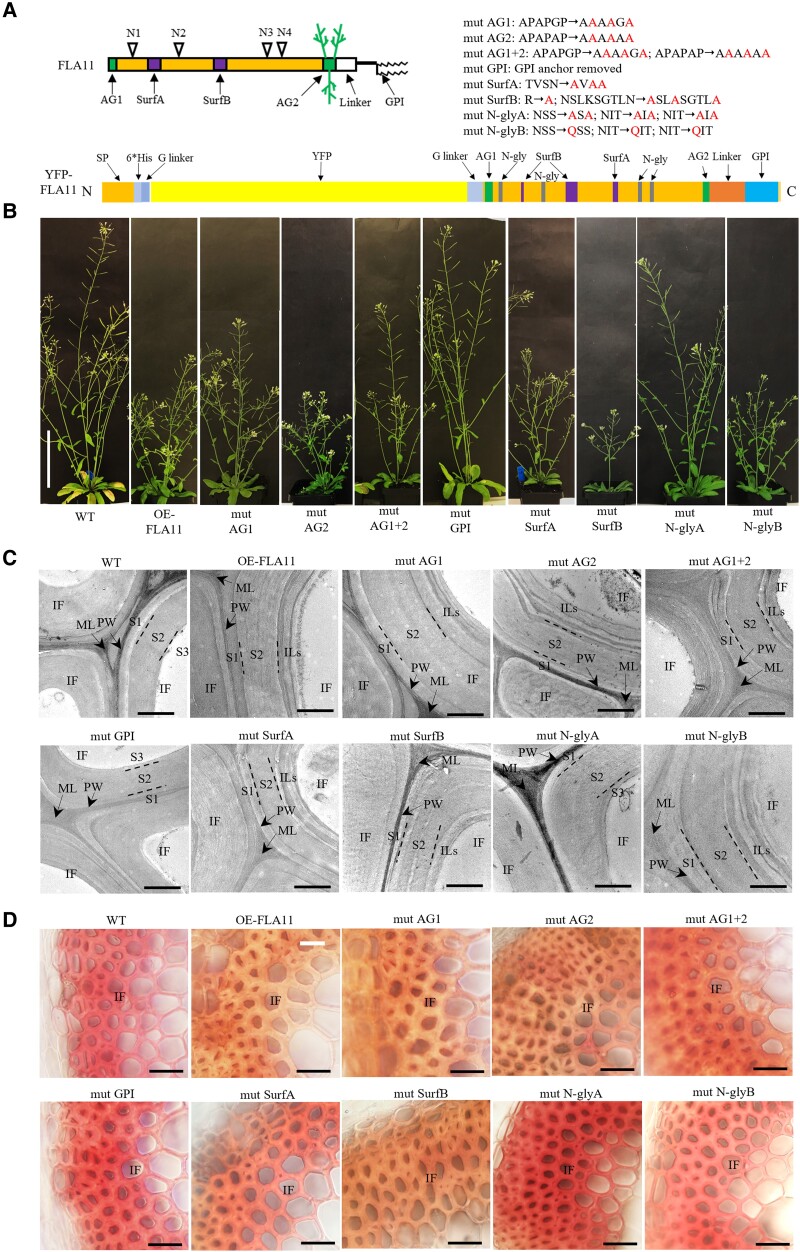
Phenotypes of mature Arabidopsis plants and IFs in WT, OE-FLA11, and mutant variants. **A)** Schematic representation of FLA11 and YFP-FLA11 protein structure with posttranslational modifications and introduced FLA11 domain mutations. Changes are indicated in the text. AG1 and AG2, arabinogalactan motif 1 and 2; *N*-gly, *N*-glycosylation motif; GPI, glycosylphosphatidylinositol; Surf A and B, predicted protein surface regions in FAS1 domain. **B)** Representative images of plants at growth stage 6.5 (Boyes et al. 2001) of WT, OE-FLA11, and OE-FLA11 mutant variants with a 1TC in the T2 generation. *proFLA11* promoter was used for all OE-FLA11 and OE-FLA11 mutant variants transgene lines. **C)** TEM imaging of transverse sections at 1 cm above the stem base of plants at stage 6.9 (Boyes et al. 2001). IF common to the SCWs shows 3 layers (S1, S2, and S3) in WT. Increased IF SCW thickness and occurrence of multiple internal layers (ILs) are observed in 1TC OE-FLA11 and similar wall phenotypes occur in lines with mutations in AG1, AG2, AG1 + 2, SurfA, SurfB, and *N*-glyB. IF SCWs of lines with mutations in GPI and *N*-glyA show similar organization to WT. **D)** Stage 6.9 plant stems were hand sectioned at 1 cm from the base and stained with Mäule reagent which stains S-lignin-rich IF cell walls a pink/red color in WT and lines with mutations in GPI and *N*-glyA plant stems, and red-brown staining indicating lower S/G-lignin ratio in lines that show similar stem phenotypes to OE-FLA11. PW, primary wall; ML, middle lamella. Scale bar = 10 cm in **B)**, 1 *µ*m in **C)**, and 20 *µ*m in **D)**.

FLA11 is a chimeric AGP predicted to have a single FAS1 domain with 4 *N*-glycosylation motifs, 2 Pro-rich regions containing AG glycan motifs that direct *O*-glycosylation, and a GPI anchor (Fig. 1A). Knockout mutants for *fla11* in Arabidopsis display subtle stem defects, including a slight reduction in height and in some cases, collapsed xylem (Persson et al. 2005; MacMillan et al. 2010). In contrast, overexpression of FLA11 (*proFLA11*::YFP-FLA11; OE-FLA11, Fig. 1B) has been shown to trigger early SCW development, increased interfascicular fiber (IF) wall thickness and lignin content (with lower S/G lignin ratio), and reduced stem length (Ma et al. 2022). Transformation of OE-FLA11 into either wild-type (WT) or *fla11* mutant showed upregulated *FLA11* transcript levels and a reduced plant stem length phenotype compared to WT and *fla11* (Supplemental Fig. S1). Since some variability has been observed in *fla11* phenotypes (Persson et al. 2005; MacMillan et al. 2010), the OE-FLA11 phenotypes in a WT background were used as the basis to investigate the functional domains of FLA11 and gain insight into the mechanism(s) of FLA11 function during SCW development.

A suite of mutated variants of the OE-FLA11 constructs were generated (Fig. 1A; Supplemental Fig. S2). These included mutations in the AG glycomotifs that direct *O*-glycosylation at either the N terminus (mutAG1), the C-terminal AG region (mutAG2), or both regions (mutAG1 + 2) and variants with the GPI anchor signal sequence removed (mutGPI). In addition, mutations within the FAS1 domain included mutation of 2 predicted surface regions (mutSurfA and mutSurfB), mutation of 3 predicted *N*-glycosylation motifs within the FAS1 domain by replacing NXS/T with AXA (mut*N*-glyA, a nonconservative mutation that will prevent *N*-glycosylation or binding-site function) and replacing NXS/T with QXS/T (mut*N*-glyB, a conservative mutation that should retain function if a binding site) (Supplemental Fig. S2; Fig. 1A).

Plants containing the YFP-FLA11 constructs were verified by sequencing of the introduced transgenes and visualization of YFP fluorescence in xylem SCWs of seedling primary roots (Supplemental Fig. S3). At least 3 independent transformed lines of each mutant variant were used for further analysis. *FLA11* transcript levels in stems were analyzed by RT-qPCR and showed that all transgenic plants had 3- to 9-fold higher expression of *FLA11* than WT (Supplemental Fig. S4A). Protein blot analysis showed all fusion proteins expressed in comparable amounts (Supplemental Fig. S4B). Both OE-FLA11 and OE-FLA11mutAG1 showed sharp bands around 60 kDa, the expected size of HIS-YFP-FLA fusion protein, and a smeared band around 110 kDa that likely corresponds to AG glycosylated proteins (Supplemental Fig. S4B). OE-FLA11mutAG2, OE-FLA11mutAG1 + 2, and OE-FLA11mutGPI proteins only showed sharp bands at 60 kDa (Supplemental Fig. S4B). OE-FLA11mutSurfA, OE-FLA11mutSurfB, OE-FLA11mut*N*-glyA, and OE-FLA11mut*N*-glyB all showed both sharp and smeared bands (Supplemental Fig. S4B). The sharp bands in OE-FLA11mut*N*-glyA and OE-FLA11mut*N*-glyB had an obvious size shift (∼5 kDa) to a smaller size, indicating that FLA11 is likely *N*-glycosylated (Supplemental Fig. S4B).

All OE-FLA11 mutation transgenic plants were phenotypically compared to WT and OE-FLA11 to determine which mutant constructs were still able to induce OE-FLA11 effects. Plants that displayed similar phenotypes to OE-FLA11 plants included OE-FLA11mutAG1, OE-FLA11mutSurfA, and OE-FLA11mut*N*-glyB (Fig. 1B). Mutation of both AG motifs and *N*-glyA plants showed an intermediate primary stem length phenotype as compared to WT and OE-FLA11 plants (Fig. 1B; Table 1). The OE-FLA11mutGPI plants did not show obvious differences from WT (Fig. 1B; Table 1). The OE-FLA11mutAG2 and OE-FLA11mutSurfB plants displayed significantly shorter stem-length phenotypes than OE-FLA11 (Fig. 1B; Table 1). OE-FLA11 has previously been shown to have increased wall thickness in stem IF (Ma et al. 2022). Transmission-electron microscopy (TEM) imaging showed that the OE-FLA11 mutant lines that showed similar phenotypes to OE-FLA11 have a similar increase in IF wall thickness and multiple inner IF wall layers (Fig. 1C). These phenotypes were not observed in OE-FLA11mutGPI and OE-FLA11mut*N*-glyA plants, similar to WT plants (Fig. 1C).

**Table 1.** Measurement of fiber length, wall thickness, crystalline cellulose content, and lignin content in stems at growth stage 6.9^a^ of WT, OE-FLA11, and mutant variants^b^

Genotype	Stem length (cm)	Fiber length (*µ*m)	IF wall thickness (*µ*m)	Cellulose (% AIR)	Lignin (% AIR)
WT	43.5 ± 1.3*	714 ± 137*	1.41 ± 0.32*	45.36 ± 4.30*	14.24 ± 1.87*
OE-FLA11	**23.8 ± 1.7**	**498 ± 134**	**1.97 ± 0.43**	**37.24 ± 2.40**	**19.44** ± **1.59**
mut AG1	33.4 ± 4.7*	**485 ± 124**	**2.07 ± 0.49**	43.84 ± 3.48	**17.80** ± **2.20**
mut AG2	14.8 ± 2.8*	429 ± 78*	1.75 ± 0.20*	29.50 ± 1.77*	**24.50** ± **3.99**
mut AG1 + 2	36.6 ± 3.7*	554 ± 132*	**1.78 ± 0.45**	47.17 ± 2.71*	**18.78** ± **3.11**
mut GPI	44.7 ± 2.2*	633 ± 158*	1.59 ± 0.18*	43.39 ± 4.24	13.72 ± 2.07*
mut SurfA	**24.9 ± 1.4**	537 ± 112*	**1.83 ± 0.39**	**40.23 ± 3.37**	**17.60** ± **0.28**
mut SurfB	14.8 ± 2.3*	426 ± 106*	1.63 ± 0.29*	31.63 ± 2.10*	**23.21** ± **3.23**
mut *N*-glyA	37.0 ± 3.6*	613 ± 138*	1.71 ± 0.33*	39.88 ± 2.73	15.63 ± 2.26
mut *N*-glyB	**29.0 ± 5.6**	**507 ± 137**	**1.96 ± 0.34**	**37.79 ± 2.97**	**23.25** ± **4.06**

Bold indicates a statistically significant difference compared to WT, and asterisk indicates a statistically significant difference compared to OE-FLA11; *P* < 0.05 using Student's *t-*test.

Growth stages as outlined in Boyes et al (2001).

Transgenic plants with 1TC were used. Data are shown as mean ± Sd. *N* = 9 plants from 3 independent transformed lines.

Stem cellular organization, fiber length, IF wall organization and thickness, crystalline cellulose content, and lignin content and composition were investigated in OE-FLA11 mutant variants (Fig. 1, C and D; Table 1; Supplemental Fig. S5). Given the severity of the OE-FLA11 line, traits were investigated in plants with 1 transgene copy (1TC), as these display lower gene expression levels and milder phenotypes than 2TC plants and enabled more developmentally equivalent stages to be compared (Ma et al. 2022). Transverse sections at 1 cm above the stem base of mature plants were stained with Toluidine blue O, for overall anatomy, phloroglucinol-HCl, and Mäule stains to visualize lignin content and composition. Compared to WT, wall thickness and lignin composition in IF walls of all mutants, except for mutGPI and mut*N*-glyA plants, were altered and similar to OE-FLA11 (Fig. 1D; Supplemental Fig. S5). The analysis of fiber length showed highly significant decreases in base stems of OE-FLA11 and lines with mutations in AG1, AG2, SurfB, and *N*-glyB plants. Moderate decreases in fiber length were observed in lines with mutations in AG1 + AG2, GPI, SurfA, and *N*-glyA compared to WT (Table 1). Wall thickness in IF cells shows highly significant increases compared to WT in lines with mutations in AG1, AG2, AG1 + AG2, SurfA, and *N*-glyB (Table 1). Moderate increases in lines with mutations in GPI, *N*-glyA, and SurfB were observed compared to WT (Table 1).

OE-FLA11 mutGPI and *N*-glyA variants that showed similar stem length and tissue organization phenotypes to WT had no significant change in lignin content (Table 1). Crystalline cellulose content was decreased in OE-FLA11 and lines with mutations in AG2, SurfA and B regions, and *N*-glyB plants compared to WT (Table 1). In contrast, no significant difference in crystalline cellulose content was found in lines with mutations in AG1, AG1 + 2, GPI, and *N*-glyA stems compared to WT (Table 1).

In summary, OE-FLA11 mutGPI and mut*N*-glyA lose the ability to induce OE-FLA11 phenotypes. OE-FLA11 mutAG1 plants display intermediate phenotypes between OE-FLA11 and WT, and OE-FLA11 mutAG2 had enhanced OE-FLA11 phenotypes.

### FLA11 is released from the PM and deposited into the SCW

**Figure 2.**
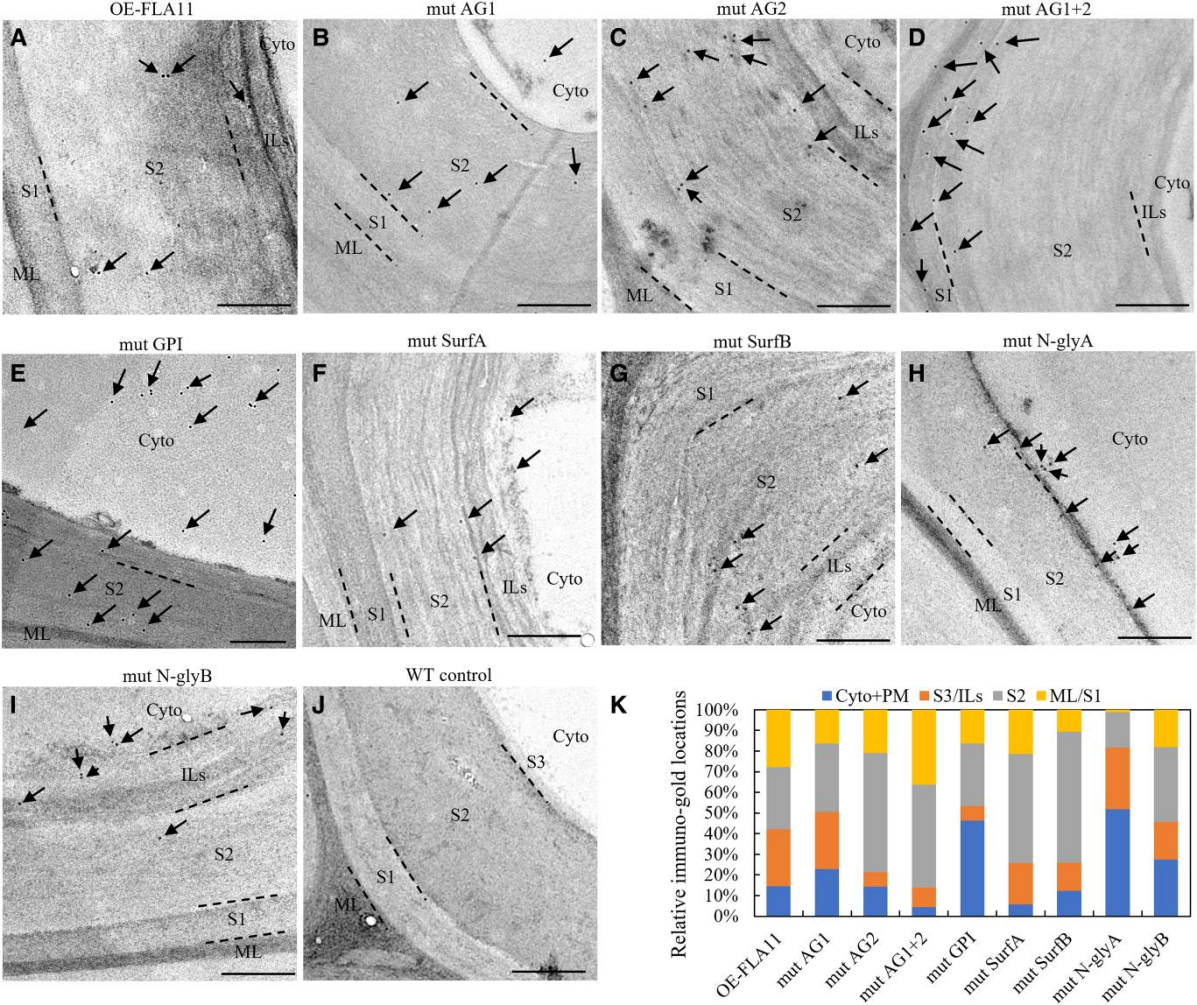
Subcellular location of HIS-YFP-FLA11 and mutant variants in IF cells at the base region of stems. TEM immuno-labeling detection of HIS-tagged FLA11 in ultrathin (∼80 nm), transverse sections at 1 cm from the stem base of stage 6.9 (Boyes et al. 2001) plants with 1TC. Arrows indicate gold particles. In IF cells of OE-FLA11 plants **A)** immuno-gold labeling is largely found in the SCWs with some label present at other locations, likely cytoplasm (cyto) and the plasma brane (PM). HIS-YFP-FLA11 signals were found mostly in the SCWs of IF cells in lines with mutations in AG1 **B)**, AG2 **C)**, AG1 + 2 **D)**, SurfA **F)**, SurfB **G)**, *N*-glyB **I)**, and WT control **J)**. In the AG1 + 2 mutation line **D)**, immuno-gold labeling was found mostly in the middle lamella, SCW S1 layer, and outer S2 layer. In mutGPI and mutN-glyA plants, the immuno-gold labeling was present in both the cytoplasm/PM and SCWs, with less gold labeling within the SCWs compared to non-mutated FLA11 **E) and H)**. **K)** Quantification of immuno-gold signals. *proFLA11* promoter was used for all OE-FLA11 and OE-FLA11 mutant variants transgene lines. Scale bar = 500 nm. ML, middle lamella; S1, S2, and S3, SCW layers S1, S2, and S3; ILs, multiple internal layers of SCWs.

The AG glycans and GPI anchor are predicted to facilitate the transport and/or location of FLA11 through the endomembrane system to the outer leaflet of the PM facing the apoplast. Phenotypes of FLA11 mutant variants either lacking glycomotifs in both the AG1 and AG2 domains or the GPI anchor signal sequence suggest that these are important for FLA11 function during SCW development. To determine if any of the introduced mutations influence FLA11 subcellular location and/or secretion, TEM was used to investigate the location of FLA11 variants in SCWs of IFs in stably transformed Arabidopsis plants. In addition to YFP, a HIS tag was included in all constructs (Supplemental Fig. S2) and was used for immuno-gold TEM labeling. In OE-FLA11 1TC lines, immuno-gold labeling was mostly in SCWs with minor amounts of signal in cytoplasm/PM (Fig. 2, A and K). Similar signal distribution patterns were found in lines with mutations in AG1, AG2, SurfA, SurfB, and *N*-glyB (Fig. 2, B, F, G, I, and K). In contrast, the immuno-gold labeling in mutAG1 + 2 plants was found in the middle lamella, SCW S1 layer, and outer S2 layer (Fig. 2, D and K). In mutGPI and mut*N*-glyA plants, the immuno-gold labeling was present in both the cytoplasm/PM and SCWs, with less gold labeling within the SCWs compared to non-mutated FLA11 (Fig. 2, E, H, and K).

In summary, the majority of FLA11 was found located at the SCW with a minor component in the cytoplasm/PM. Disruption of AG glycan, GPI anchor, and *N*-glycosylation motifs within the FAS1 domain influences the trafficking/targeting of FLA11 into SCWs.

### FLA3, FLA11, and FLA12 are functionally distinct

**Figure 3.**
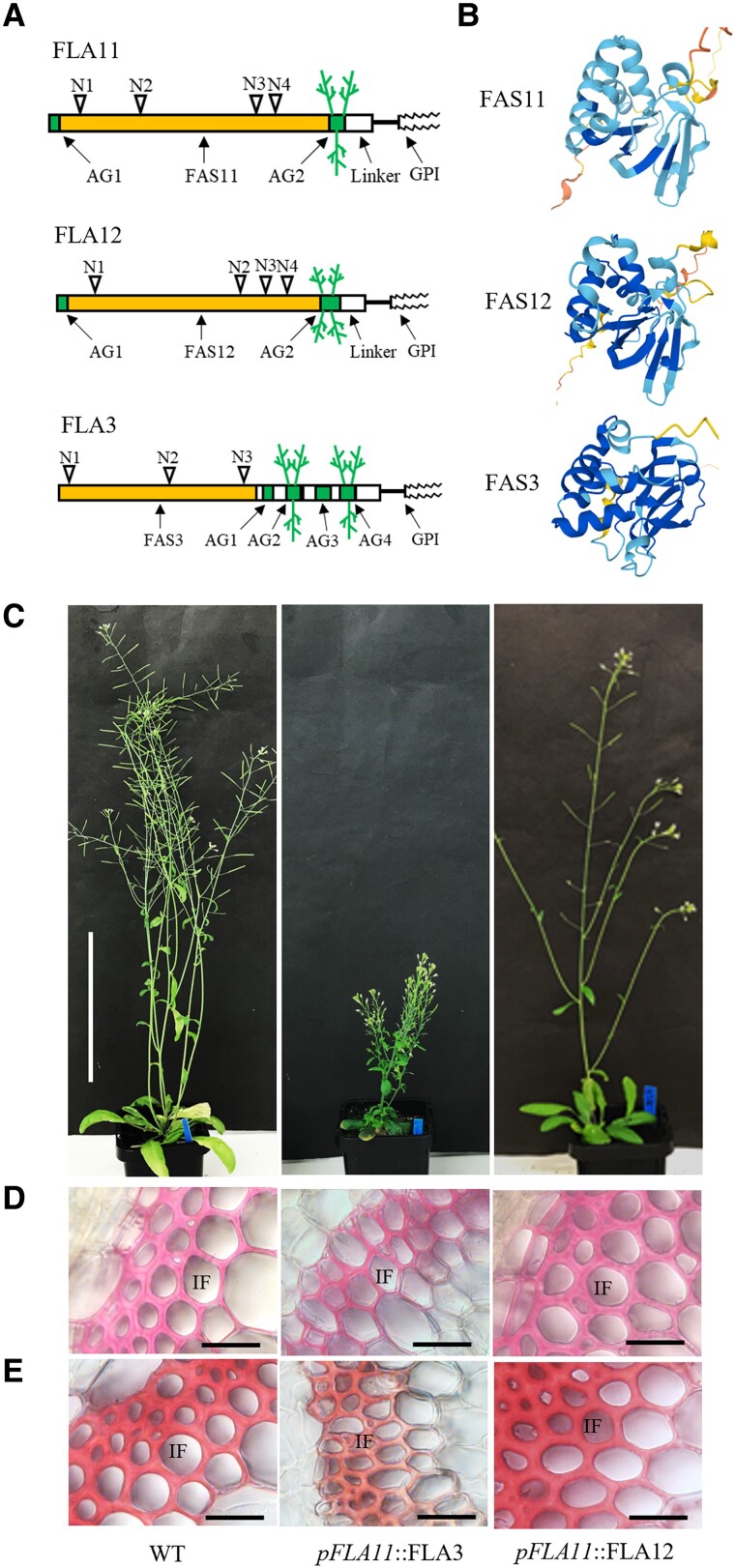
Comparison of FLA11, FLA12, and FLA3 domain structures and SCW phenotypes. **A)** Schematic representation of FLA11, FLA12, and FLA3 protein structures with predicted posttranslational modifications. **B)** Modeling of the FAS1 domain of FLA11 (FAS11), FLA12 (FAS12), and FLA3 (FAS3) using AlphaFold (Jumper et al. 2021) suggests that FAS11 and FAS12 have similar structures that differ from FAS3. **C)** Representative images of stage 6.9 (Boyes et al. 2001) plant morphology of WT, *pFLA11*::FLA3, and *pFLA11*::FLA12. **D)** and **E)** Histological analyses of IFs at the base of stems. Sections taken from fresh stems of stage 6.9 plants at 1 cm from the base stained with either phloroglucinol-HCl **D)** or Mäule stain **E)** to show cellular morphology and lignin composition. Stems of *pFLA11*::FLA3 plant show thinner walls compared to WT. Stems of *pFLA11*::FLA12 plant show similar wall thickness and lignin composition to WT. Scale bar = 10 cm in **C)** and 20 *µ*m in **D)** and **E)**.

Our results showed that mutation of the FAS1 domain and GPI anchor in FLA11 disrupt OE-FLA11 SCW phenotypes. Mutation of theAG2 domain suggests that it may act as a regulatory region. The majority of FLAs found in plant proteomes throughout evolution consist of AG, FAS1, and GPI anchor domain structures yet play diverse biological roles (Shafee et al. 2020). To investigate what domain structure(s) provide specificity for FLA11 to function in SCW development, FLA11, FLA12, and FLA3 were used for domain swap and functional comparison (Supplemental Fig. S6). FLA11, FLA12, and FLA3 all predict protein structures with a single FAS1 domain, 2-4 AG glycan domains, and a C-terminal GPI anchor (Fig. 3A). FLA12 is the most closely related to FLA11 and also functions in SCW development (MacMillan et al. 2010; Ma et al. 2022). FLA3 is a group C FLA predominantly expressed in reproductive organs and not suggested to be involved in SCW development (Johnson et al. 2003; Li et al. 2010). Consistent with previous studies, the predicted FAS1 structures (Fig. 3B) showed that FAS11 and FAS12 are similar (Type O) and both are different from FAS3 (Type F) (Shafee et al. 2020; Jumper et al. 2021).

The functional equivalence of FLA11, FLA12, and FLA3 was examined when expressed under the *FLA11* promoter (Supplemental Fig. S6). Protein blot analysis showed that the introduced constructs were expressed (Supplemental Fig. S7A). A molecular weight smeared band between 110 and 160 kDa was observed for *pFLA11*::FLA3 and corresponded to the increased number of AG glycomotifs (Supplemental Fig. S6). Interestingly, protein blots of *pFLA11*::FLA12 showed a smeared band of approximately 75 kDa, smaller than observed for FLA11 and suggesting differences in the amount or size of AG glycosylation between these proteins (Supplemental Fig. S8A). Expressing HIS-YFP-FLA3 in SCWs driven by *proFLA11* (*pFLA11*::FLA3) resulted in plants with reduced stem length and weaker stems compared to WT (Fig. 3, C–E). Stem transverse sections of *pFLA11*::FLA3 showed disrupted development of SCWs, including thinner walls and less lignin in both xylem vessels (XVs) and IFs compared to WT and OE-FLA11. Unlike OE-FLA11, the introduction of *proFLA11*::HIS-YFP-FLA12 into WT (*pFLA11*::FLA12) did not result in plant growth phenotypes (Fig. 3, C–E; Supplemental Fig. S7, B–D). Histological analyses of transverse sections at 1 cm from the stem base of *pFLA11*::FLA12 transgenic plants at stage 6.9 stained with either Toluidine blue O, phloroglucinol-HCL, or Mäule stains showed similar tissue organization, XV and IF morphologies, and lignin composition to WT (Fig. 3, C–E; Supplemental Fig. S7, B–D).

In summary, these results showed that although FLA11, FLA12, and FLA3 share similar domain organization, they are not functionally equivalent. Ectopic expression of FLA3 in stems resulted in suppression of SCW development, whereas FLA12 showed no obvious changes in stem SCW development. FLA11 appears to be the predominant regulator of stem SCW initiation and development.

### FAS1 domain is functionally equivalent between FLA11 and FLA12, and AG2 is a major determinant for the different functions and subcellular locations between FLA11 and FLA12

**Figure 4.**
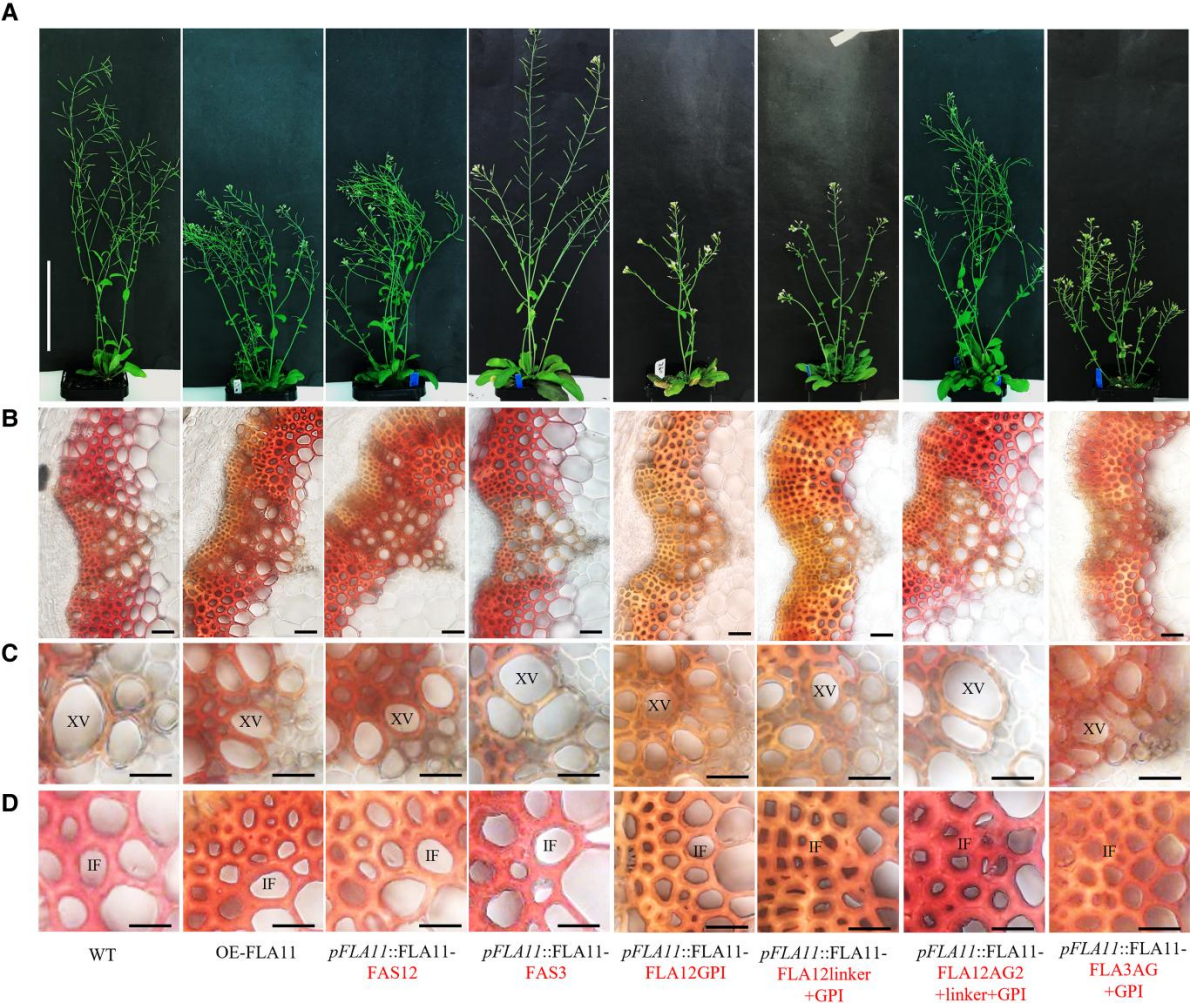
Comparison of FAS1, AG2, linker, and GPI domain functions for FLA11 regulation of SCW development. **A)** Representative images of stage 6.9 (Boyes et al. 2001) plant morphology of WT, OE-FLA11, *pFLA11*::FLA11-FAS12, *pFLA11*::FLA11-FAS3, *pFLA11*::FLA11-FLA12GPI, *pFLA11*::FLA11-FLA12linker + GPI, *pFLA11*::FLA11-FLA12AG2 + linker + GPI, and *pFLA11*::FLA11-FLA3AG + GPI (see Table 2 for stem length quantification, Supplemental Fig. S6 for a schematic representation of proteins). **B)–D)** Histological analyses of the base of stems **B)**, cell morphology of xylem vessels (XVs) **C)**, and IFs and wall lignin composition **D)**. Sections taken from fresh stems of stage 6.9 plants at 1 cm from the base were stained with Mäule reagent. Stems of OE-FLA11, *pFLA11*::FLA11-FAS12, *pFLA11*::FLA11-FLA12GPI, *pFLA11*::FLA11-FLA12linker + GPI, and *pFLA11*::FLA11-FLA3AG + GPI plant show smaller XV diameters compared to WT, and IFs with red/brown color compared to WT with pink/red color. Other plants show similar XV diameter and IF lignin composition compared to WT. Scale bar = 10 cm in **A)**, 20 *µ*m in **B)**, and 10 *µ*m in **C)** and **D)**. The domains swapped are indicated in the text.

The specificity of the FAS1 region for the FLA11 function was investigated by swapping the FAS1 domain of FLA11 (FAS11, Type O) with the FAS1 domain of FLA12 (FAS12, Type O) and FLA3 (FAS3, Type F) (see Supplemental Fig. S6). Protein blotting analysis showed that both *pFLA11*::FLA11-FAS12 and *pFLA11*::FLA11-FAS3 proteins were expressed in comparable amounts (Supplemental Fig. S8A). Smeared bands at 110 kDa were observed in both OE-FLA11 and *pFLA11*::FLA11-FAS12 proteins, and not in *pFLA11*::FLA11-FAS3 (Supplemental Fig. S8A). Phenotypic analysis showed that *pFLA11*::FLA11-FAS12 plants have similar phenotypes to OE-FLA11 plants, including reduced stem length (Fig. 4A; Table 2), smaller base stem XV diameters (Fig. 4, B and C), reduced S-lignin content in base stem IF walls (Fig. 4D), increased stem lignin content (Table 2), and decreased crystalline cellulose content (Table 2) compared to WT. *pFLA11*::FLA11-FAS3 plants show similar phenotypes to WT (Fig. 4; Table 2). These data suggest that FAS11 and FAS12 are functionally equivalent, and different from FAS3.

**Table 2.** Comparison of OE-FLA11-FAS1, AG2, and GPI domain swap plants^a^ stem length, lignin content, and crystalline cellulose content at growth stage 6.9^b^

Plant lines	Stem length (cm)	Lignin content (% AIR)	Crystalline cellulose content (% AIR)
WT	39.8 ± 3.4	12.0 ± 0.7	45.7 ± 3.9
OE-FLA11	**23.0 ± 1.0**	**13.5** ± **0.4**	**33.3** ± **1.9**
*pFLA11*::FLA11-**FAS12**	**24.8** ± **13.2**	**14.1** ± **2.3**	**40.3** ± **1.5**
*pFLA11*::FLA11-**FAS3**	40.8 ± 5.7	11.7 ± 3.0	47.7 ± 3.7
*pFLA11*::FLA11-**FLA12GPI**	**23.3** ± **1.0**	**14.6** ± **2.5**	**38.6** ± **5.1**
*pFLA11*::FLA11-**FLA12linker + GPI**	**27.4** ± **9.5**	**14.9** ± **1.8**	**40.6** ± **2.7**
*pFLA11*::FLA11-**FLA12AG2 + linker + GPI**	40.9 ± 2.3	11.2 ± 2.7	47.1 ± 3.5
*pFLA11*::FLA11-**FLA3AG + GPI**	**25.3** ± **0.6**	**15.9** ± **2.8**	**39.5** ± **9.3**

Bold indicates a statistically significant difference, *P* < 0.05 using Student's *t-*test. Bold text indicates domains swapped; see Supplemental Fig. S6 for a schematic representation of domain swaps.

Transgenic plants with 1TC were used. Data are shown as mean ± Sd. *N* = 9 plants (for stem length) or 3 plants (for lignin and cellulose content) from 3 independent transformed lines.

Growth stages as outlined in Boyes et al. (2001).

The specificity of AG2 glycans and GPI anchor signal sequence for FLA11 function(s) were experimentally investigated by swapping FLA11 AG2 motif, GPI anchor signal sequence, and disordered linker region with the equivalent region from either FLA12 or FLA3 (see Supplemental Fig. S6). Analysis of *FLA11* transcripts by RT-qPCR showed that *pFLA11*::FLA11-FLA12GPI, *pFLA11*::FLA11-FLA12linker + GPI, *pFLA11*::FLA11-FLA12AG2 + linker + GPI, and *pFLA11*::FLA11-FLA3AG + GPI plants all had 3- to 6-fold increased *FLA11* transcript levels in stems compared to WT (Supplemental Fig. S8B). Protein blot analysis showed that all fusion proteins were present at comparable levels and with the expected sizes (Supplemental Fig. S8C). As expected by the larger number of predicted AG glycomotifs in FLA3, the *pFLA11*::FLA11-FLA3AG + GPI showed a higher molecular weight smeared band between 110 and 160 kDa and was consistent with protein blots of *pFLA11*::FLA3 (Supplemental Fig. S8C). Consistent with *pFLA11*:FLA12 that appears to have a lower amount of glycosylation than FLA11 (Supplemental Fig. S7A), minimal glycosylation of *pFLA11*::FLA11-FLA12AG2 + linker + GPI appears to occur with no obvious higher molecular weight 110 kDa band (Supplemental Fig. S8C).

All domain swap plants were used for phenotypic analysis, and *pFLA11*::FLA11-FLA12GPI, *pFLA11*::FLA11-FLA12linker + GPI, and *pFLA11*::FLA11-FLA3AG + GPI plants showed reduced stem length (Fig. 4A; Table 2), smaller XV diameter (Fig. 4, B and C), lower S-lignin in IF walls (Fig. 4D), increased stem lignin content, and decreased cellulose contents (Table 2) compared to WT, similar to OE-FLA11. Interestingly, *pFLA11*::FLA11-FLA12AG2 + linker + GPI plants displayed similar plant growth and SCW phenotypes as WT (Fig. 4; Table 2).

TEM immuno-labeling was used to investigate whether AG2 affects FLA11 subcellular location. In *pFLA11*::FLA11-FLA12linker + GPI plant stem IF walls, immuno-gold labeling was mostly found in SCWs with minor amounts of signal in the PM/cytoplasm (Supplemental Fig. S9, A and C). In *pFLA11*::FLA11-FLA12AG2 + linker + GPI plant stem IF walls, immuno-gold labeling was mostly retained in the cytoplasm, with less signal in SCWs (Supplemental Fig. S9, B and C). In summary, these data suggest that (i) FLA11, FLA12, and FLA3 GPI anchor signal sequences are functionally equivalent, and (ii) AG2 regions of FLA11 and FLA3 are functionally equivalent, whereas the FLA12 AG2 region impacts FLA11 function and subcellular location.

## Discussion

The role of FLAs in regulating many aspects of plant development indicates functional specificity; yet, the structural features conveying these roles have remained elusive (Ma et al. 2022). FLA members belong to large gene families, with most diploid eudicots and monocots having around 20 members (Johnson et al. 2003, 2017a, 2017b). FLAs vary in their domain types and organization, as well as both overlapping and specific functions of closely related members having been identified (Shi et al. 2003; Li et al. 2010; MacMillan et al. 2010; Johnson et al. 2011, 2017a, 2017b; Liu et al. 2020; Shafee et al. 2020; Ma et al. 2022). FLAs are multidomain glycoproteins and many are predicted to be located at the PM/wall interface, tethered by GPI anchors. The current study is the most comprehensive investigation to date attempting to dissect the different FLA domains and their impact on the subcellular location and biological function.

PM microdomain location is likely required for the FLA11 function. GPI anchors are predicted to be added to many FLAs including the Group A subclass to which FLA11 belongs (Shafee et al. 2020). GPI anchors are proposed to direct proteins to specific PM microdomains/lipid rafts, which may be important for assisting protein interactions with receptors for signaling (Tapken and Murphy 2015; Zavaliev et al. 2016; Yeats et al. 2018). GPI anchoring of FLA11 at the PM, a location that appears to be transient before releasing into the wall, was shown to be required for the OE-FLA11 phenotypes (Fig. 1). This suggests that FLA11 signaling partners, involved in regulating the initiation of SCW development and compositional changes, are co-located in close proximity to the PM, likely PM microdomains. Support for GPI-anchored proteins regulating receptor-like kinase (RLK) location and activity is shown by LORELEI and LORELEI-LIKE GPI-anchored proteins which are essential for PM localization of the RLK FERONIA that has been implicated in cell wall sensing in multiple pathways (Li et al. 2015; Liu et al. 2016). A GPI anchor has also been shown to be sufficient for targeting specific cellular locations, as shown by the fusion of GPI anchors from 2 plasmodesmata-located proteins CALLOSE BINDING 1 (PDCB1) and β-1,3-GLUCANASE (PdBG2) to 2 non-plasmodesmata-localized proteins, AGP4 and LIPID TRANSFER PROTEIN1 (LTGP1) (Zavaliev et al. 2016). The presence of PDCB1 and PdBG2 GPI anchors directed the accumulation of AGP4 and LTGP1 to plasmodesmata (Zavaliev et al. 2016; Yeats et al. 2018). Apart from the well-recognized roles of GPI anchor in regulating protein secretion, our results also showed that OE-FLA11 mutGPI proteins have no obvious AG glycosylation (Supplemental Fig. S4), suggesting a role of GPI anchor for FLA11 AG glycosylation. Further experiments are required to confirm this relationship and explore what specific aspects GPI anchor plays in regulating AG glycan synthesis. One possibility is supported by previous research, which showed that disruption of GPI anchor synthesis impacts the trafficking and anchoring of GPI-anchored proteins (Bernat-Silvestre et al. 2021).

Our data indicated a potential functional relationship between AG1 and AG2 glycosylated regions in FLA11 for deposition into SCWs as the release of FLA11 from PM into SCWs was shown to be influenced by AG glycosylation. AG glycans are highly complex structures and their roles in regulating plant growth and development are not well understood (Ma et al. 2018). Previous studies have shown that an AG glycan chain, AMOR, can play a role in regulating cell–cell signaling during reproduction (Mizukami et al. 2016) and an AGP, APAP1, cross-links xylans and pectins in primary walls, thereby modulating the matrix phase network (Tan et al. 2013). A cell surface AG peptide, AGP21, shown to regulate root hair fate relies on brassinosteroid (BR) signaling and disruption of AG glycans by AG motif mutation, β-Glc-Yariv treatment, or AG glycan synthesis eliminates AGP21 function and leads to contiguous root hair phenotype (Borassi et al. 2020). To date, functional studies of FLA domains have only been undertaken for AtFLA4, also known as AtSOS5 due to a salt overly sensitive phenotype when mutated (Shi et al. 2003). The AG region in AtFLA4/SOS5 was shown to influence secretion to the PM/wall; however, it did not affect its biological function during salt stress (Xue et al. 2017). Our data showed a weak phenotype in OE-FLA11mutAG1 + AG2 plant and FLA11 proteins located at S1 and outer S2 layers in SCWs. We have unveiled the distinction between different AG regions, with AG1 minimally if at all glycosylated and AG2 shown to lead to a higher molecular weight “smear” on protein blots consistent with containing AG *O*-glycans (Poon et al. 2012; Xue et al. 2017). Domain swap experiments showed that glycosylation of the AG2 region is required for FLA11 function and to distinguish it from FLA12. We cannot exclude the possibility that the OE-FLA11-FLA12AG2 + linker + GPI construct has artificially affected the function of the FAS1 domain considering that OE-FLA11mutAG2 was able to induce OE-FLA11 phenotypes. Whether the AG glycomotif is sufficient to direct different size and type of AG *O*-glycans remain to be determined. Our domain swap results suggest that the FAS1 domain can affect AG glycans, suggesting that the surrounding domains may also play a role in regulating AG glycosylation. The AG glycan structure could potentially interact with wall polysaccharides to control the deposition/secretion of FLA11 into the wall, similar to APAP1 structures (Tan et al. 2013). Alternatively, a function for the GlcA residues on the AG glycans being involved in calcium complexing and signaling was demonstrated (Lamport and Varnai 2013; Lopez-Hernandez et al. 2020). It remains unclear which specific AGPs (based upon the protein backbone) are involved in these interactions, in what tissues, and if members with fewer AG glycosylation motifs, such as the FLAs, have similar roles (Johnson et al. 2003, 2017a, 2017b). Future experiments undertaking AG glycan structure analysis and modification of the terminal residues will aid our understanding of these important regions.

FLA11 may play a role as a molecular scaffold for a cell surface sensing cluster. FAS1 domains are evolutionary ancient domains found in both animals and plants (Bastiani et al. 1987; Elkins et al. 1990; Johnson et al. 2003). FAS1-containing proteins in animals include periostin, proposed to act as a scaffold mediating extracellular matrix formation that can interact with a variety of proteins to activate signaling (Kii and Ito 2017). The specific role(s) of FAS1 domains in plants have not been well characterized. Further work is required to confirm FLA11 interacting partners and the requirement of the FAS1-domain for associations. Either dynamics (movement within the PM) or endocytosis of the protein clusters is common for cell sensing and the role of AGP in interacting with rare earth elements and regulating clathrin-mediated endocytosis has been demonstrated (Wang et al. 2019). *N*-glycosylation is found in almost all FAS1 domains of FLAs and a domain deletion experiment suggested that AtFLA4 trafficking can be regulated by *N*-glycosylation (Xue et al. 2017). Our *N*-glyB domain mutation data showed that *N*-glycosylation within the FAS1 domain of FLA11 had no obvious effects in regulating FLA11 trafficking. The role of *N*-glycosylation within the FAS1 domain in regulating the trafficking of different FLAs requires further investigation. Dissection of FLA domain–structure–function relationships in this study shows that the function of FAS1 domain-containing proteins in Arabidopsis requires AG glycosylation and GPI anchor attachment, and these influence the location and interactions in the polysaccharide-rich extracellular environments. This work provides the foundations that underpin the exploration of the signaling pathways of plant FLAs and insight into the functional associations of other chimeric AGPs.

## Materials and methods

### Plant materials and growth conditions

Arabidopsis (*Arabidopsis thaliana*) (Col-0), *fla11* mutant (SALK_046976), and OE-FLA11/FLA12/FLA3 domain mutation/deletion/swap variants plants were grown under long day (16 h light/8 h dark) conditions at 22/18 °C in controlled environment rooms.

### Vector construction and Agrobacterium (Agrobacterium tumefaciens) transformation

A pGreenII0179 vector backbone (Hellens et al. 2000) was reconstructed by replacing *CaMV 35S* with a native *proFLA11* promoter for constructing *proFLA11*::His-YFP-FLA11 vector as used in a previous study (Ma et al. 2022). Supplemental Fig. S2 and Supplemental Tables S1 and S2 provide the list of vectors and primers used for FLA11 domain mutation/deletion. FLA11, FLA12, and FLA3 domain swaps vectors and primers can be found in Supplemental Fig. S6 and Supplemental Tables S3 and S4. NEBuilder HiFi DNA Assembly kit (NEW ENGLAND Biolabs) was used to construct vectors according to the manufacturer's instructions. All vectors were confirmed by sequencing and then transformed into *Agrobacterium* strain AGL1.

Arabidopsis plants were transformed using the flower dip method (Clough and Bent 1998). Plants were then screened with hygromycin (35 mg/L). The number of insertions and transgene copies (TC) was predicted based on segregation ratios in the T2 and T3 generation. Although the segregation ratio alone is not sufficient to confirm the TC numbers, RT-qPCR analyses in our previous work (Ma et al. 2022) confirmed the gene expression differences in 1TC and 2TC plants that were selected based on the segregation ratios. Lines with 70% to 85% survival ratio (3:1) were selected and further used for checking YFP signals in primary root vascular tissues. Plants from at least 3 independent T2 lines were transferred into the soil for further growth and phenotypic analysis. If transgenic plants from all 3 T2 lines showed a dwarf phenotype similar to OE-FLA11, their progeny (T3) were used for further analysis. In the segregating T3 generation, plants with a moderate dwarf phenotype (1TC plants) were used for phenotypic analysis and data collection. Transgenic plants with no T2 lines showing a dwarf phenotype (at least 3 independent lines) were taken to the T3 generation and lines with a 100% survival ratio (2TC) were used for phenotypic analysis.

### Histological analysis

Fresh stems were hand-sectioned and stained with either Toluidine blue O, phloroglucinol-HCl, or Mäule stain to visualize cell walls according to the methods outlined in Mitra and Loqué (2014) with an Olympus BX53 microscope under a bright field. At least 3 plant stems from 3 independent transformed lines were sectioned and measured for tissue organization analyses. The data shown represent mean ± Sd. Student’s *t-*test was used for significance analysis with *P* < 0.05.

YFP signals were detected in whole-mount primary roots of 10-d-old seedlings using Zeiss LSM 780 laser scanning confocal microscope (excitation lasers at 514 nm with 5% strength, emission at 570 nm, collection at 519–621 nm, detector gains 912.9). At least 3 plants from 3 independent transgenic lines were imaged with 1 representative image shown.

### TEM and immuno-labeling

A 2-mm region at the base of stems at growth 6.9 were chemically fixed, dehydrated, and embedded in LR white according to the method outlined in Wilson and Bacic (2012). Thin sections (∼80 nm) were acquired for antibody labeling and post-stained (Wilson and Bacic 2012). For antibody labeling, samples were incubated with anti-6x-His tag monoclonal (Invitrogen, # MA1-21315) at 1:100 dilutions for 1 h at room temperature, and then overnight at 4 °C. Samples were then washed and incubated with goat anti-mouse 18 nm gold-conjugated secondary antibody (Jackson Immuno Research #115-215-166) at 1:20 dilutions for 1 h at room temperature. Detection of ultrastructure, HIS-YFP-FLA11, and HIS-YFP-FLA12 subcellular location was performed on 2 biological replicates using 2 technical replicates. Grids were imaged using a Jeol 2100 EM equipped with a Gatan Orius SC 200 CCD camera. Gold signals were quantified manually from TEM images and data were shown as mean ±Sd.

### Fiber length and wall thickness analyses

To measure stem fiber length, Arabidopsis base stems (2 cm length of stem from the base) were incubated with glacial acetic acid and hydrogen peroxide (v/v 1:1) for 12 h at 80 °C (Wang et al. 2020). Fibers were stained with Gram's safranin solution and imaged under a bright field using an Olympus BX53 microscopy. Each measurement contained approximately 200 fiber cells from 2 plants from 2 independent transformed lines. Data represent mean ± Sd. Student’s *t-*test was used for significance analysis with *P* < 0.05.

For the measurement of IF wall thickness, TEM images were used. Primary IF cell layers closest to the pith were used for quantification with 2 biological samples from 2 independent transformed lines. At least 10 cells were used for quantification. Data represent mean ± Sd. Student’s *t-*test was used for significance analysis with *P* < 0.05.

### Measurement of crystalline cellulose and lignin content

Arabidopsis stems were harvested and alcohol-insoluble residue (AIR) was prepared (Pettolino et al. 2012). The Updegraff method was used for the measurement of crystalline cellulose content (Updegraff 1969). The acetyl bromide method was used to detect lignin content according to Chang et al. (2008). Three biological replicates from 3 independent transformed lines were measured. Data represent mean ± Sd. Student’s *t-*test was used for significance analysis with *P* < 0.05.

### RT-qPCR analysis

RNA was extracted from the stem tissue at growth stage 6.0 using the Spectrum Plant Total RNA Kit (Sigma #STRN250). Complementary DNA was synthesized using SuperScript IV Reverse Transcriptase (Invitrogen). RT-qPCR was conducted to measure the transcript levels of *FLA11* using a relative quantitative method (Livak and Schmittgen 2001), with 2 or 3 biological replicates from 2 or 3 independent transformed lines, and 3 technical replicates in a QUANTSTUDIO 5 Real-Time System with 384 wells (Thermo Fisher, Waltham, MA, USA) using PowerUp SYBR Green Master Mix (2X) Universal (A25742; Thermo Fisher) in 10 *μ*L reactions. Transcript levels were normalized against the housekeeping gene *ACT2*. RT-qPCR primers were as used in Ma et al. (2022) and can be found in Supplemental Table S5.

### Protein enrichment and blotting

Arabidopsis seedlings (10 d old) were harvested for total protein preparation and GFP-trap enrichment. Total proteins were extracted using extraction buffer containing 100 mM Tris-HCl at pH 8.8, 150 mM NaCl, 1 mM EDTA, 10% (v/v) glycerol, and 1 × cOmplete protease inhibitor cocktail (Sigma, #11697498001). Total proteins were enriched using a GFP-trap (ChromoTek, #gtma20) according to the manufacturer's instructions at 4 °C for 1 h with 1% (w/v) *n*-Dodecyl-β-D-maltoside (DDM). Denatured proteins were used for SDS-PAGE and protein blots analysis and detected with anti-GFP (Invitrogen, #MA5-15256). Two biological replicates from 2 independent transformed lines were performed.

### Accession numbers

Sequence data from this article can be found in the GenBank/EMBL data libraries under accession numbers: *FLA11 (AT5G03170), FLA12 (AT5G60490), FLA3 (AT2G24450),* and *ACT2 (AT3G18780)*.

## Supplementary Material

kiad097_Supplementary_Data

## Data Availability

All data generated or analysed during this study are included in this published article (and its supplementary information files).

## Supplemental data

The following materials are available in the online version of this article.

**Supplemental Figure S1**. RT-qPCR and phenotypic analyses of WT OE-FLA11 and *fla11* OE-FLA11 transgenic plants compared with wild-type (WT) plants.

**Supplemental Figure S2**. YFP- FLA11 fusion protein amino acid sequence and schematic representation of FLA11 mutant variants.

**Supplemental Figure S3**. Visualization of YFP in wild-type (WT) Arabidopsis plants stably transformed with OE-FLA11 and its mutant variants.

**Supplemental Figure S4**. RT-qPCR and protein blotting analysis of OE-FLA11 and mutant variant plants.

**Supplemental Figure S5**. Histological analyses of interfascicular fibers (IFs) at the base of stems of WT, OE-FLA11, and OE-FLA11 mutant variant plants with 1 transgene copy (1TC).

**Supplemental Figure S6**. Schematic representation of FLA domain swaps and YFP-FLA12 and FLA3 protein sequences.

**Supplemental Figure S7**. Comparison of FLA12 and FLA3 domain swap fusion proteins and histological analyses of xylem vessels (XVs) at the base of stems of wild-type (WT), *pFLA11*::FLA3, and *pFLA11*::FLA12.

**Supplemental Figure S8**. Comparison of FLA11, FLA12, and FLA3 domain swap fusion proteins.

**Supplemental Figure S9**. Subcellular location of HIS-YFP-FLA11-FLA12linker-FLA12GPI and HIS-YFP-FLA11-FLA12AG2-FLA12linker-FLA12GPI in interfascicular fiber (IF) cells at the base region of stems.

**Supplemental Table S1**. List of vectors used for FLA11 domain mutation and deletion.

**Supplemental Table S2**. List of primers used for FLA11 domain mutation/deletion vector constructions.

**Supplemental Table S3**. List of vectors used for domain swaps.

**Supplemental Table S4**. List of primers used for domain swap vector constructs.

**Supplemental Table S5**. List of primers used for RT-qPCR.
