# Correction to: Distinct functions of FASCICLIN-LIKE ARABINOGALACTAN PROTEINS relate to domain structure

**DOI:** 10.1093/plphys/kiad284

**Published:** 2023-05-17

This is a correction to: Yingxuan Ma, Thomas Shafee, Asha M Mudiyanselage, Julian Ratcliffe, Colleen P MacMillan, Shawn D Mansfield, Antony Bacic, Kim L Johnson, Distinct functions of FASCICLIN-LIKE ARABINOGALACTAN PROTEINS relate to domain structure, *Plant Physiology*, Volume 192, Issue 1, May 2023, Pages 119–132, https://doi.org/10.1093/plphys/kiad097

In the originally published version of this manuscript, there was a typographical error in the title, whereby ‘FASCICLIN-LIKE’ was given as ‘FASCILIN-LIKE’.

This error has been corrected.

