# Supplementary material for: Distinct functions of FASCICLIN-LIKE ARABINOGALACTAN PROTEINS relate to domain structure
Source: Plant Physiol. 2023 Feb 17;192(1):119–32. doi: 10.1093/plphys/kiad097 (PMC10152678; doi:10.1093/plphys/kiad097)
Supplement: kiad097_Supplementary_Data [file kiad097_supplementary_data.pdf]

Supplemental Data for

Distinct functions of FASCILIN-LIKE ARABINOGALACTAN PROTEINS relate to domain structure

Yingxuan Ma[1,2], Thomas Shafee[2], Asha M Mudiyanselage[2], Julian Ratcliffe[2], Colleen P MacMillan[3], Shawn D Mansfield[4], Antony Bacic[2,5], Kim L Johnson[2,5*]

* Corresponding author: Kim L Johnson

Email: k.johnson@latrobe.edu.au

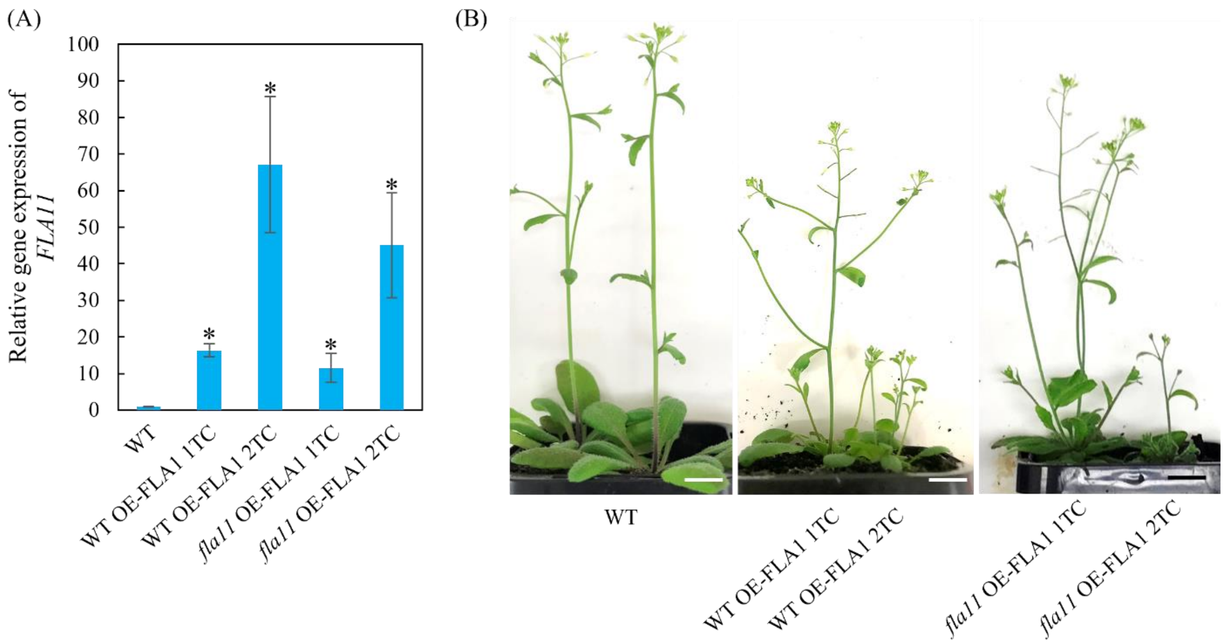

Supplemental Figure S1. RT-qPCR and phenotypic analyses of WT OE-FLA11 and *fla11* OE-FLA11 transgenic plants compared with wild-type (WT) plants. (A) RT-qPCR analysis of stage 6.0 (Boyes et al., 2001) plant stems showed *FLA11* transcript levels relative to *ACT2* up-regulated in both WT OE-FLA11 and *fla11* OE-FLA11 transgenic plants compared to wild type (WT). Data shown are average values ± SD (n ≥ 3 plants from three independent transformed lines). Asterisks indicate a significant difference compared to WT plants according to Student's *t*-test (*, $P < 0.05$). **(B)** Phenotypic analysis of stage 6.1 (Boyes et al., 2001) WT OE-FLA11 and *fla11* OE-FLA11 transgenic plants with one (1TC) or two transgene copies (2TC) and compared to WT. 2TC plants with higher *FLA11* expression levels displayed shorter plant stem length than one transgene copy (1TC) lines. *FLA11* expression levels and plant stem phenotypes were comparable in OE-FLA11 plants with either WT or *fla11* background. Scale: 1 cm.

(A) MATSRTFIFSNLFIFFLVIATTYGPGHHHHHHGGGGSMVSKGEELFTGVVPILVELDGDVNGHKFSVSGE
GEGDATYGKLTLKLICTTGKLPVPWPTLVTTLGYGLQCFARYPDHMKQHDFFKSAMPEGYVQERTIFFK
DDGNYKTRAEVKFEGDTLVNRIELKGIDFKEDGNILGHKLEYNYNSHNVYLTADKQKNGIKANFKIRHN
IEDGGVQLADHYQQNTPIGDGPVLLPDNHYLSYQSALSKDPNEKRDHMVLLEFVTAAGITLGMDELYK
GGGGSGGGGSPRQAPAPGPSGPTNITAILEKAGQFTLFIRLLKSTQASDQINTQLNSSSSNGLTVFAPTDNA
FNSLKSGTLNSLSDQQKVQLVQFHVLPTLITMPQFQTVSNPLRTQAGDGQNGKFPLNITSSGNQVNITTG
VVSATVANSVYSDKQLAVYQVDQVLLPLAMFGSSVAPAPAPEKGGSVSKGSASGGDDGGDSTDSSDAE
RTGFGFGIRITTVAAIAASSSLWI

(B)

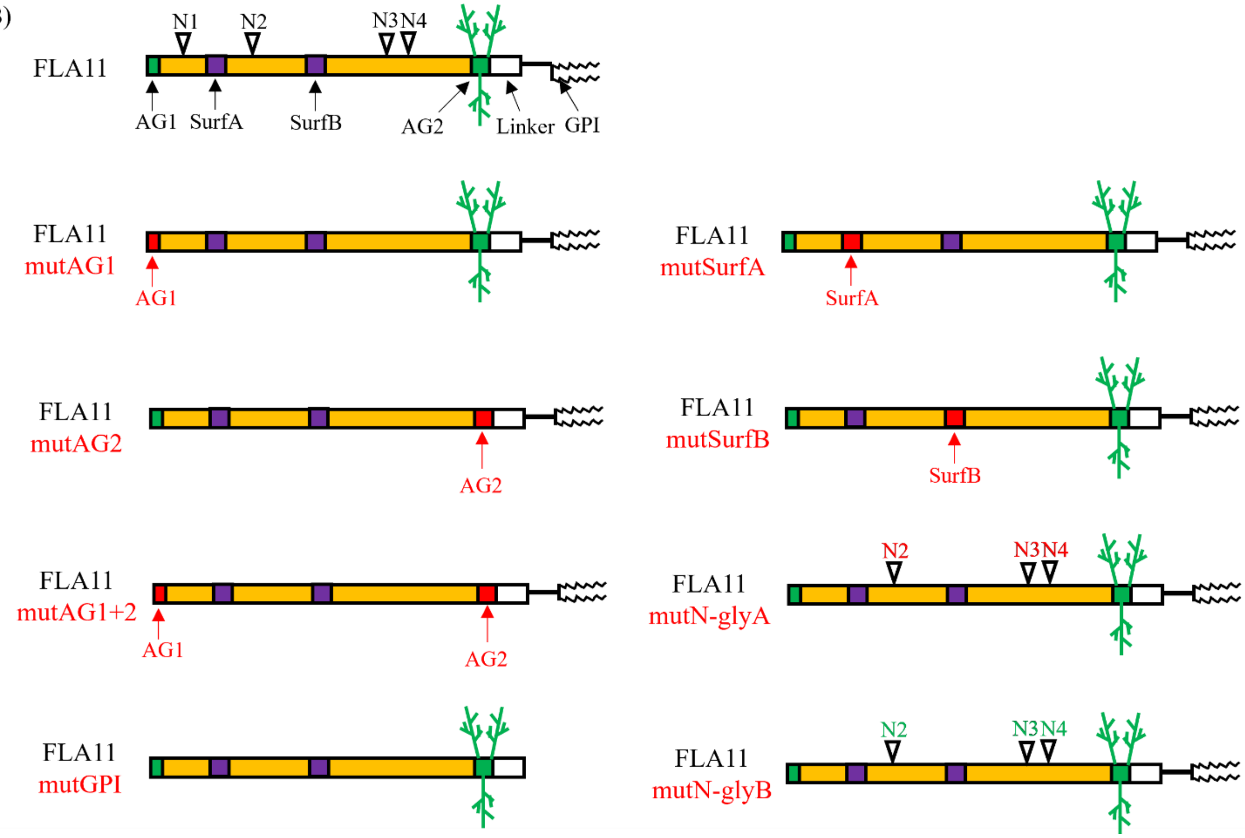

Supplemental Figure S2. YFP- FLA11 fusion protein amino acid sequence and schematic representation of FLA11 mutant variants. (A) The YFP sequence (yellow highlighted) was inserted after the FLA11 signal peptide (SP, underlined) and 6*His tag and G linker (blue), another G linker (blue) was inserted between YFP and FLA11 no SP sequence. Other predicted domains are indicated: arabinogalactan motif 1 and 2 (green), N-glycosylation motif (blue highlighted), Surf A and B (purple with grey highlighted), linker (orange), glycosylphosphatidylinositol (GPI)-anchor (black). **(B)** Schematic representation of FLA11 mutant variants.

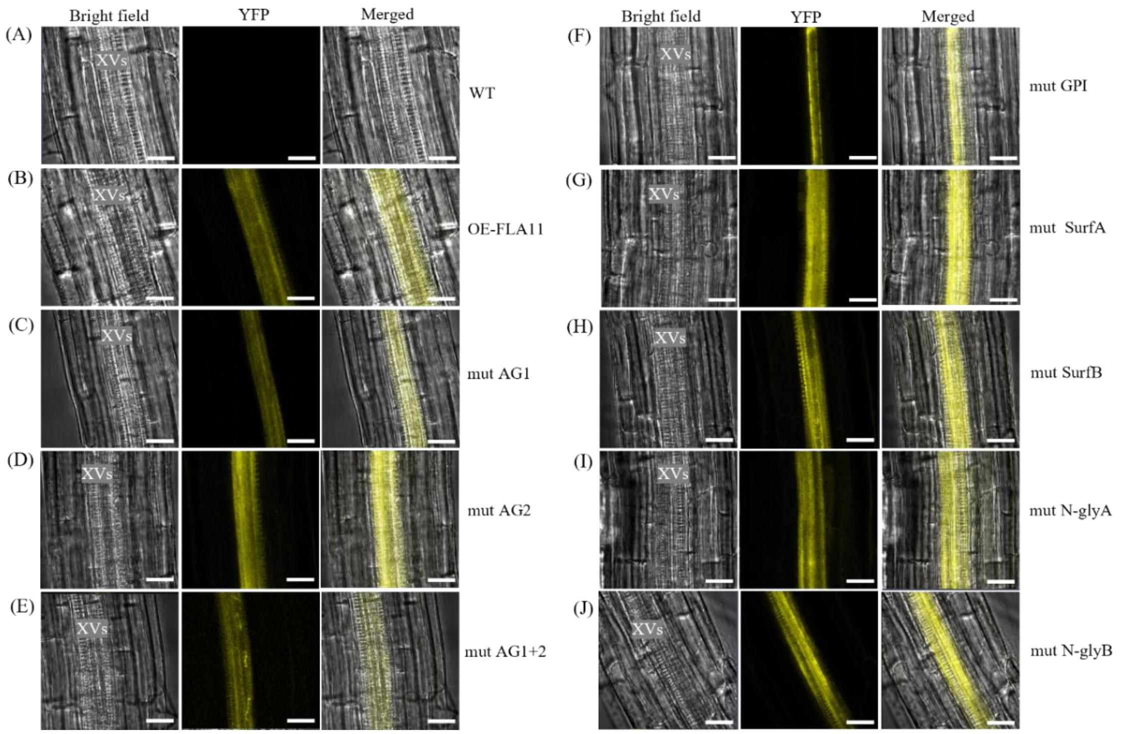

Supplemental Figure S3. Visualization of YFP in wild-type (WT) Arabidopsis plants stably transformed with OE-FLA11 and its mutant variants. YFP fluorescence signals in 10-day old seedling roots of WT **(A)**, OE-FLA11 **(B)** and lines with mutations in AG1 **(C)**, AG2 **(D)**, AG1+2 **(E)**, GPI **(F)**, SurfA **(G)**, SurfB **(H)**, N-glyA **(I)**, and N-glyB **(J)** in xylem vessels (XVs). Scale bar = 20 µm. At least three independent transformed lines of each mutant variant were used for further analysis.

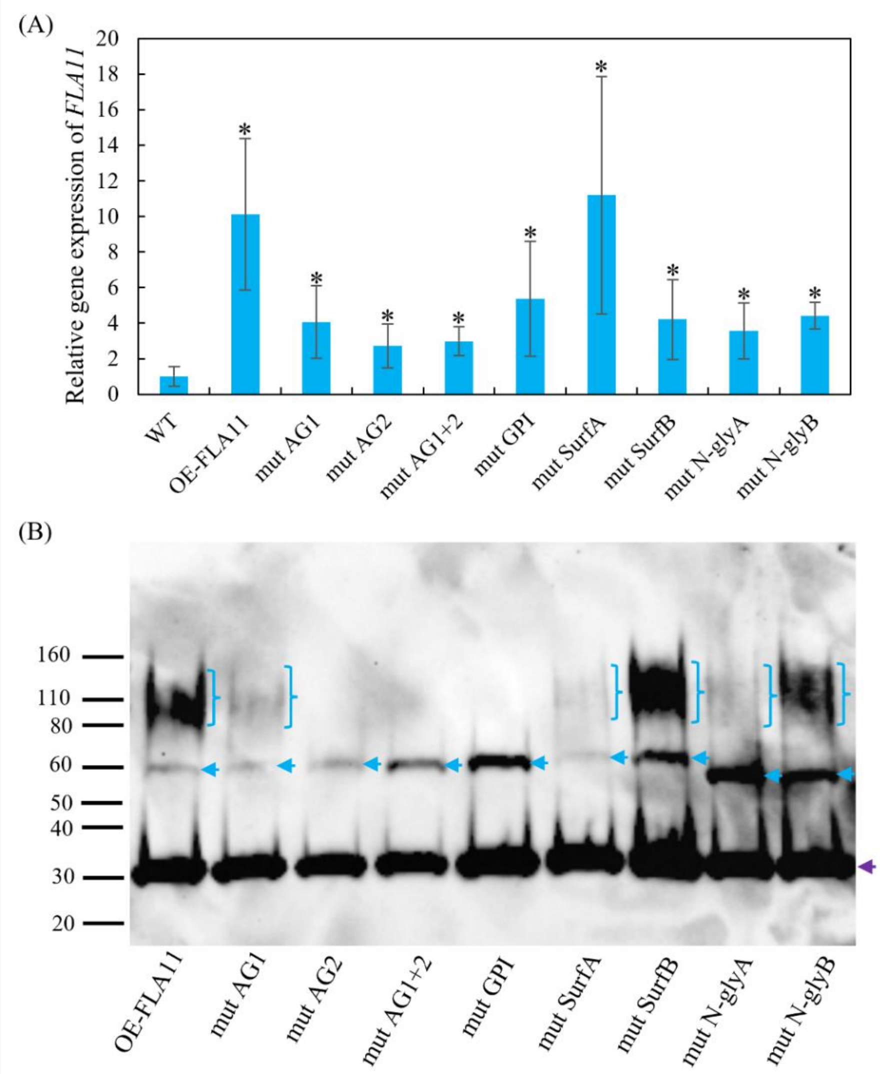

Supplemental Figure S4. RT-qPCR and protein blotting analysis of OE-FLA11 and mutant variant plants. (A) RT-qPCR results showing *FLA11* transcript levels relative to *ACT2* in wild-type (WT), OE-FLA11 and FLA11 domain mutation plants. Data shown are average values ± SD ($n \geq 3$ plants from three independent transformed lines). Asterisks indicate a significant difference compared to WT plants according to Student's *t*-test (*, $P < 0.05$). **(B)** Denatured GFP-trap enriched proteins extracted from OE-FLA11 and OE-FLA11 mutant variants 10-day-old seedlings were used for SDS-PAGE and protein blot analysis and detected with anti-YFP antibody. Blue arrows indicate YFP-FLA11/FLA11 mutant variant proteins that are un-glycosylated with MW around 60 kDa. Purple arrows indicated YFP cleaved from fusion proteins. Smeared bands of higher MW, indicated by blue brackets, likely represent proteins with AG glycosylation.

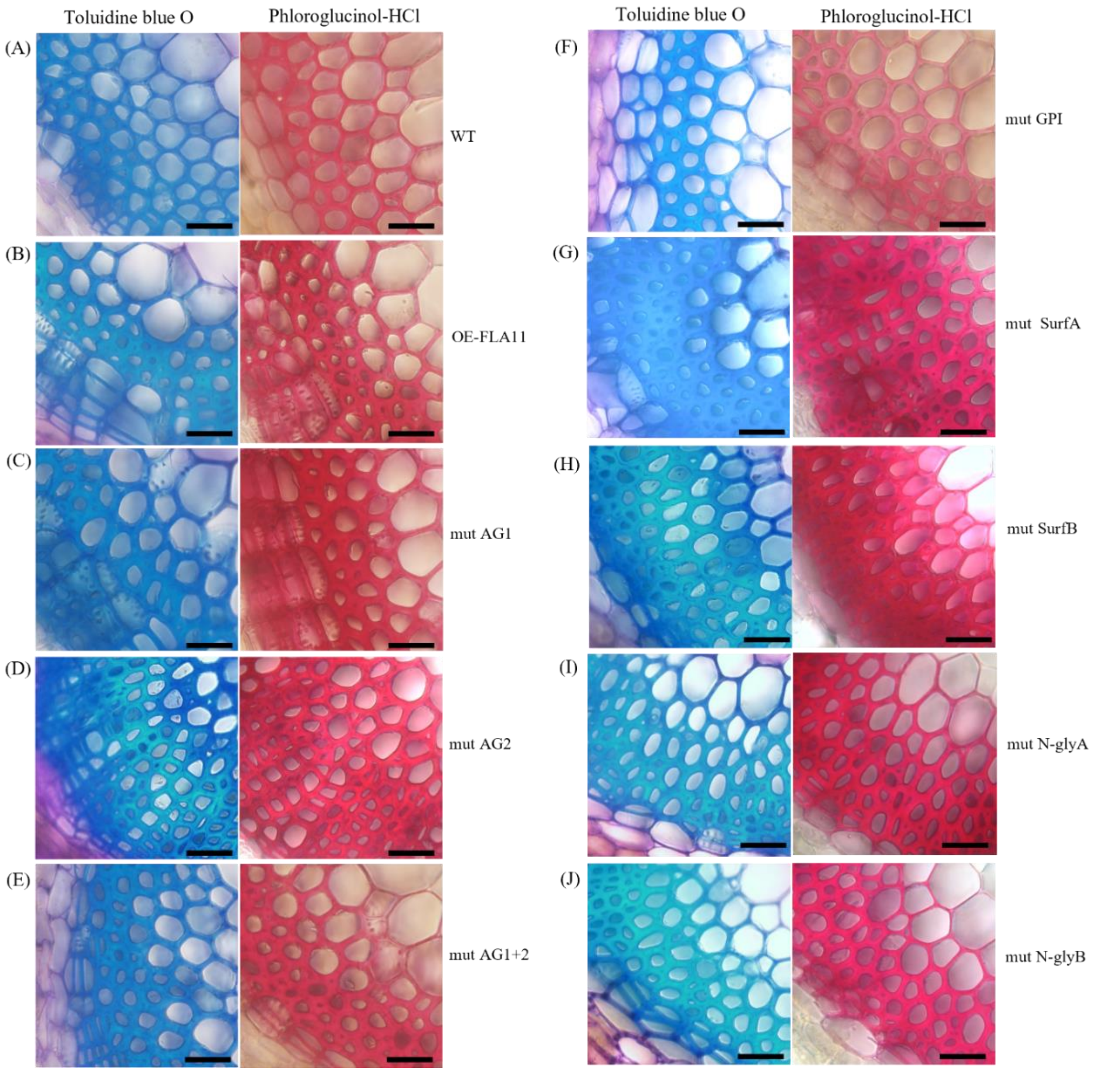

Supplemental Figure S5. Histological analyses of interfascicular fibers (IFs) at the base of stems of WT, OE-FLA11, and OE-FLA11 mutant variant plants with one transgene copy (1TC). Sections taken from fresh stems of stage 6.9 plants (Boyes et al., 2001) at 1 cm from the base were stained with either Toluidine blue O or phloroglucinol-HCl to show cellular morphology and lignin composition. Compared to WT stem IFs (**A**), OE-FLA11 (**B**) appear to have thicker IF walls. IF walls in OE-FLA11 lines with mutations in AG1 (**C**), AG2 (**D**), AG1+2 (**E**), SurfA (**G**), SurfB (**H**), and N-glyB (**J**) show similar wall thickness and lignin staining to OE-FLA11 whereas lines with mutations in GPI (**F**) and N-glyA (**I**) are more similar to WT. Scale bar = 20 µm.

(A)

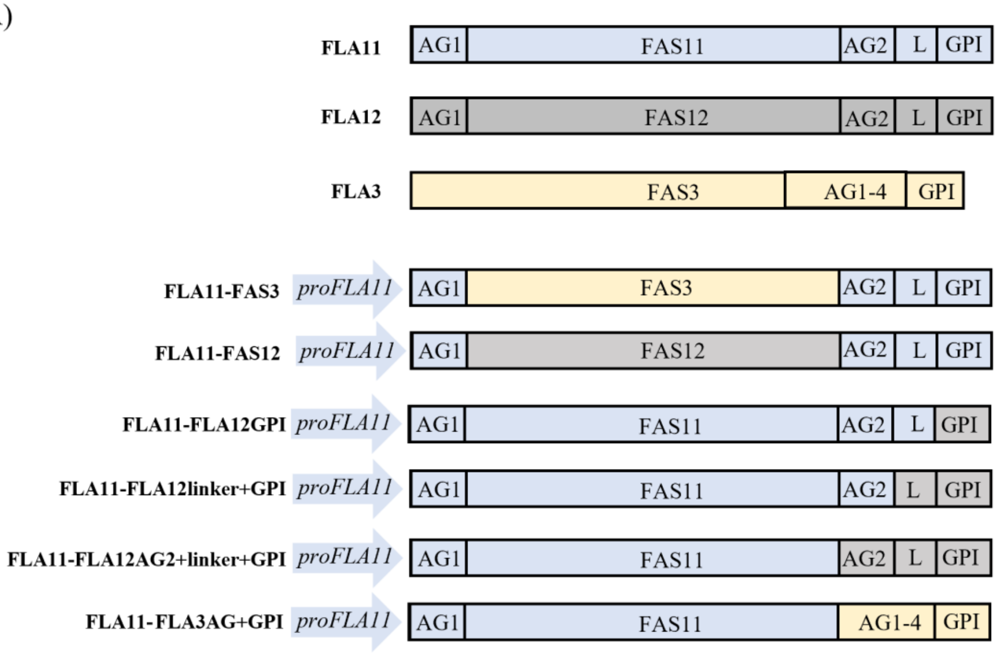

(B)

FLA12 without signal peptide:

QPSPAVAPAPPGPTNVTKILEKAGQFTVFIRLLKSTGVANQLYGQLNNSDNGITIFAPSDSSFTGLKAG
TLNSLTDEQQVELIQFHVIPSYVSSSNFQTISNPLRTQAGDSADGHFPLNVTTSGNTVNITSGVTNTTV
SGNVYSDGQLAVYQVDKVLLPQQVFDPRPPAPAPAPSVSKSKKKKDDSDSSSDDSPADASFALRNV
GSVCDAVSFCVMSVMLAWFYL

FLA3 without signal peptide:

VNITRVLEKYPEFSTMTELLAKTELTPIINKRQTITVLALNNDAIGSISGRPEEEVKNILMNHVVLDYF
DELKLKALKEKSTLLTTLYQSTGLGQQQNGFLNCTKSNGKIYFGSGVKGAPQTAEYITTVFRNPYNL
SVVQISMPIVAPGLGSPVKVPPPPPMSSPPAPSPKKGAATPAPAPADEGDYADAPPGLAPETAPASAP
SESDSPAPAPDKSGKKKMAAADEAEPPSSASNTGLSFGAVLVLGFVASFVGF

Supplemental Figure S6. Schematic representation of FLA domain swaps and YFP-FLA12 and FLA3 protein sequences. (A) Schematic representation of FLA11, FLA12 and FLA3 domain swaps. FLA11, FLA12 and FLA3 proteins shaded with blue, grey, and yellow, respectively. AG1: AG glycomotif at N-terminal. AG2: AG glycomotif closest to GPI-anchor region (GPI) at C-terminal. AG1-4: AG glycomotif 1 to 4 of FLA3, indicated in green text in (**B**). FAS11: FAS1 region of FLA11. FAS12: FAS1 region of FLA12. FAS3: FAS1 region of FLA3. **(B)** FLA12 and FLA3 protein amino acid sequences with highlighting to indicate domains/motifs. Arabinogalactan glycomotif (green), N-glycosylation motif within FAS1 domain (dark grey), and glycosylphosphatidylinositol (light blue highlighting) are indicated in the text.

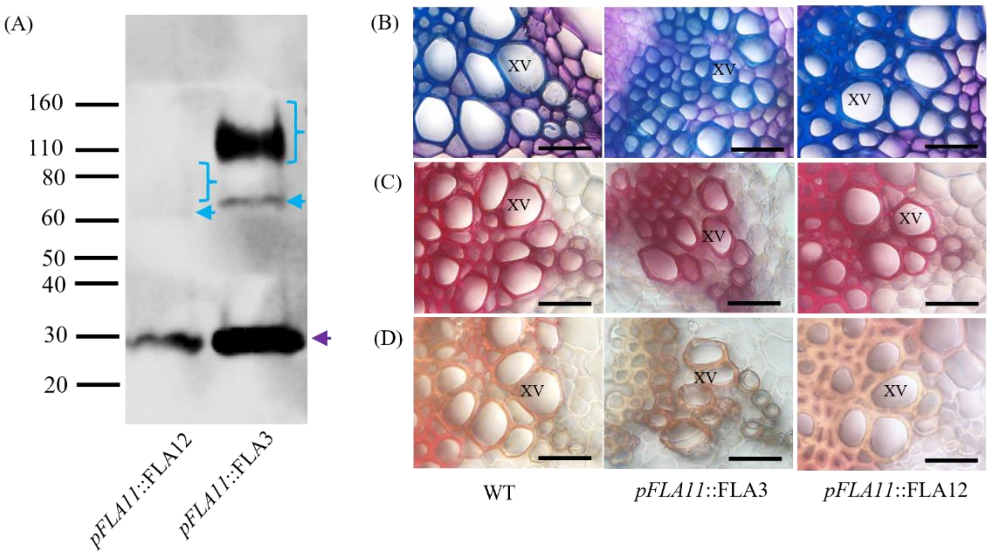

Supplemental Figure S7. Comparison of FLA12 and FLA3 domain swap fusion proteins and histological analyses of xylem vessels (XVs) at the base of stems of wild-type (WT), *pFLA11*::FLA3, and *pFLA11*::FLA12. (A) Protein blotting of GFP-trap enriched *pFLA11*::FLA12 and *pFLA11*::FLA3 from 10-day-old seedlings labelled with anti-YFP antibody. Blue arrows indicate YFP-FLA12/FLA3 and domain swap/mutation variant proteins that are un-glycosylated with MW around 60 kDa. Purple arrows indicate YFP cleaved from fusion proteins. Smeared bands of higher MW, indicated by blue brackets, likely represent proteins with AG glycosylation. **(B-D)** Sections taken from fresh stems of stage 6.9 plants (Boyes *et al*., 2001) at 1 cm from the base were stained with either Toluidine blue **(B)**, phloroglucinol-HCl **(C)** or Mäule **(D)** to show cellular morphology and lignin composition. *pFLA11*::FLA3 plant stems show smaller XV diameters compared to WT. *pFLA11*::FLA12 plant stems show similar XV diameter and lignin composition compared to WT. Scale bar = 20 µm.

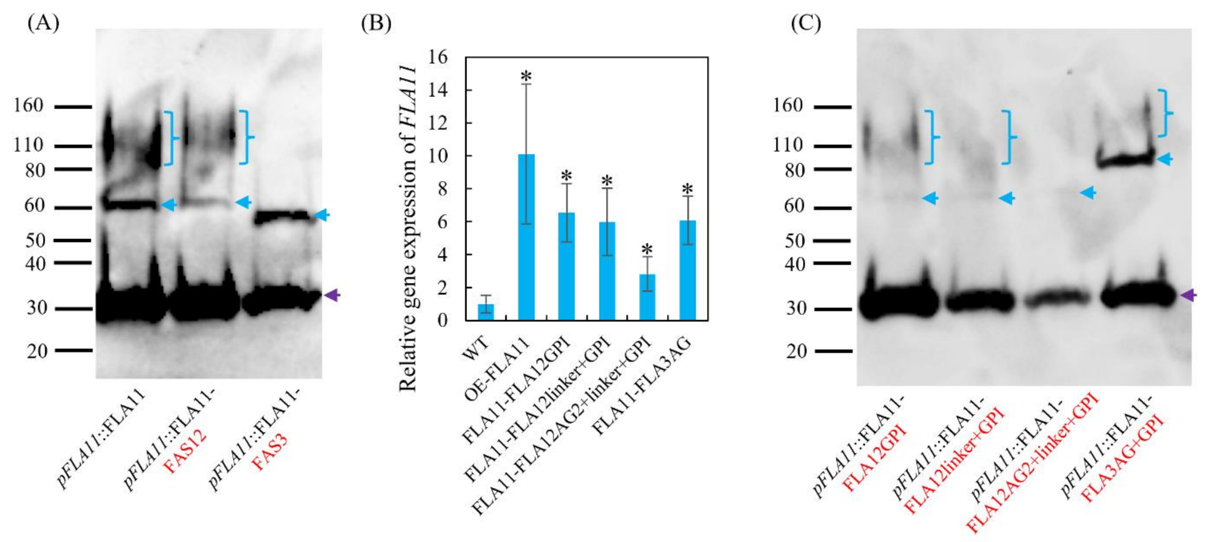

Supplemental Figure S8. Comparison of FLA11, FLA12, and FLA3 domain swap fusion proteins. (A) Protein blotting of GFP-trap enriched OE-FLA11, *pFLA11*::FLA11-FAS12, and *pFLA11*::FLA11-FAS3 from 10-day-old seedlings labelled with anti-YFP antibody. Blue arrows indicate YFP-FLA11 and domain swap/mutation variant proteins that are un-glycosylated with MW around 60 kDa. Purple arrows indicate YFP cleaved from fusion proteins. Smeared bands of higher MW, indicated by blue brackets, likely represent proteins with AG glycosylation. **(B)** *FLA11* transcript levels relative to *ACT2* in domain swap plants. Data shown are average values ± SD (n ≥ 3 plants from three independent transformed lines). Asterisks indicate a significant difference compared to WT plants according to Student's *t*-test (*, $P < 0.05$). **(C)** Protein blotting of GFP-trap enriched *pFLA11*::FLA11-FLA12GPI, *pFLA11*::FLA11-FLA12linker+GPI, *pFLA11*::FLA11-FLA12AG2+linker+GPI, and *pFLA11*::FLA11-FLA3AG+GPI from 10-day-old seedlings labelled with anti-YFP antibody.

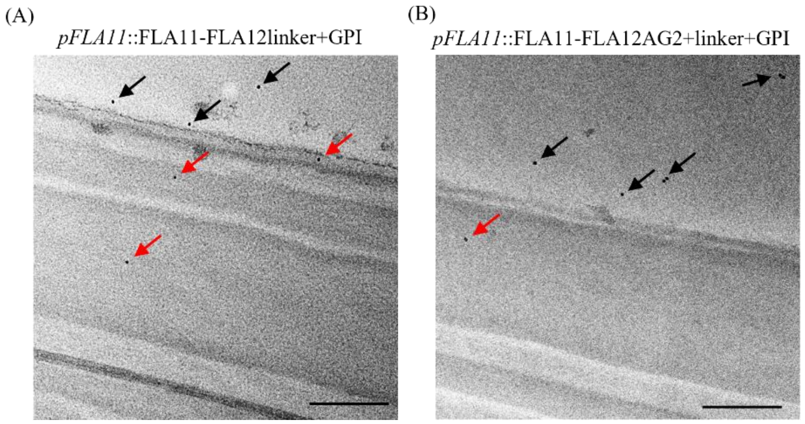

(C)

Relative immuno-gold locations (%)

	FLA11-AG2	FLA12-AG2
SCWs	67 ± 26	**26 ± 22**
Cyto+PM	33 ± 26	**74 ± 22**

Supplemental Figure S9. Sub-cellular location of HIS-YFP-FLA11-FLA12linker-FLA12GPI and HIS-YFP-FLA11-FLA12AG2-FLA12linker-FLA12GPI in interfascicular fiber (IF) cells at the base region of stems. Transmission-electron microscopy (TEM) immuno-labelling detection of HIS-tagged FLA11 in ultrathin, transverse sections at 1 cm from the stem base of stage 6.9 plants with one transgene copy (1TC). Red arrows indicate gold particles in SCWs, black arrows indicate gold particles in plasma membrane (PM)/cytoplasm (cyto). In IF cells of *pFLA11*::FLA11-FLA12linker+GPI plants **(A)** immuno-gold labelling is largely found in the SCWs with some label present at the PM/cyto. In IF cells of *pFLA11*::FLA11-FLA12AG2+linker+GPI plants **(B)** immuno-gold labelling is largely found in the cyto+PM with some label present at the SCWs. **(C)** Quantification of immuno-gold signals in SCWs and PM/cyto. Data shown as average ± SD. N = two biological replicates and two technical replicates from two independent transformed lines. Scale bar = 500 nm.

Supplemental Table S1. List of vectors used for FLA11 domain mutation and deletion.

Vector ID	Description	Purpose
YMV111	pGreen0179-*proFLA11*::spFLA11-His-YFP-FLA11	Stable transformation in *Arabidopsis*
YMV112	pGreen0179-*proFLA11*::spFLA11-His-YFP-FLA11 AG1 mutation	
YMV113	pGreen0179-*proFLA11*::spFLA11-His-YFP-FLA11 AG2 mutation	
YMV114	pGreen0179-*proFLA11*::spFLA11-His-YFP-FLA11 AG1+2 mutation	
YMV115	pGreen0179-*proFLA11*::spFLA11-His-YFP-FLA11 GPI deletion	
YMV116	pGreen0179-*proFLA11*::spFLA11-His-YFP-FLA11 SurfA mutation	
YMV117	pGreen0179-*proFLA11*::spFLA11-His-YFP-FLA11 SurfB mutation	
YMV118	pGreen0179-*proFLA11*::spFLA11-His-YFP-FLA11 N-glyA mutation	
YMV119	pGreen0179-*proFLA11*::spFLA11-His-YFP-FLA11 N-glyB mutation	

Supplemental Table S2. List of primers used for FLA11 domain mutation/deletion vector constructions.

Name	Primer sequence	Purpose
YMV111-*proFLA11*-F	ACTATAGGGCGAATTGGGTACCcagcagcgtagatcttttgagtg	Cloning *FLA11* promoter with overlaps for vector construction
YMV111-*proFLA11*-R	GAATGTTCTTGAAGTAGCCATGGtgttgtagttgttgtgtgtgtatgttg	
pGreenKpnI35S-F	CGACTCACTATAGGGCGAATTG	*FLA11* AG1 mutation vector construction
FLA11-AG Mut1_1R	agcgcctgcagctgcagcCTGcctaggTGACCCACCTC	
FLA11-AG Mut1_2F	GCTGCAGCTGCAGGCGCTTCAGGTCCAACGAACATAACC	
pGreenNotIOCS-R	CTGGAGCTCCACCGCG	
pGreenKpnI35S-F	CGACTCACTATAGGGCGAATTG	*FLA11* AG2 mutation vector construction
FLA11-AG Mut2_1R	agcgcctgcagctgcagcCTGcctaggTGACCCACCTC	
FLA11-AG Mut2_2F	GCTGCAGCTGCAGGCGCTTCAGGTCCAACGAACATAACC	
pGreenNotIOCS-R	CTGGAGCTCCACCGCG	
pGreenKpnI35S-F	CGACTCACTATAGGGCGAATTG	*FLA11* AG1+AG2 mutation vector construction
FLA11-AG Mut1_1R	agcgcctgcagctgcagcCTGcctaggTGACCCACCTC	
FLA11-AG Mut1_2F	GCTGCAGCTGCAGGCGCTTCAGGTCCAACGAACATAACC	
FLA11-AG Mut2_1R	agcgcctgcagctgcagcCTGcctaggTGACCCACCTC	
FLA11-AG Mut2_2F	GCTGCAGCTGCAGGCGCTTCAGGTCCAACGAACATAACC	
pGreenNotIOCS-R	CTGGAGCTCCACCGCG	
pGreenKpnI35S-F	CGACTCACTATAGGGCGAATTG	*FLA11* GPI deletion vector construction
YM105FLA11noGPI_R	CATATCTCATTAAAGCAGGACTCTAGATTATGAATCAGTAGAATCTCCTCCATCAT	
pGreenKpnI35S-F	CGACTCACTATAGGGCGAATTG	*FLA11* Surface A mutation vector construction
F11-SurA-Mut_1R	AGCTGCAACGGCTTGAAACTGAGGCATGGTTATGA	
F11-SurA-Mut_2F	GCCGTTGCAGCTCCTTTACGCACGCAAGCTG	
pGreenNotIOCS-R	CTGGAGCTCCACCGCG	
pGreenKpnI35S-F	CGACTCACTATAGGGCGAATTG	*FLA11* Surface B mutation vector construction
F11-SurB-Mut_1R	tgcTATGAACAATGTGAATTGACCAGC	
F11-SurB-Mut_2Fnew	CAATTCACATTGTTCATAgcaCTTCTTAAAAGCACTCAAGCCTCA	
F11-SurB-Mut_2R	cgctaaggttccggaagcgaggcttgcGAAGGCGTTATCAGTCGGG	
F11-SurB-Mut_3F	gcaagcctcgcttccggaaccttagcgTCATTGTCTGACCAACAAAAAGTTC	
pGreenNotIOCS-R	CTGGAGCTCCACCGCG	
pGreenKpnI35S-F	CGACTCACTATAGGGCGAATTG	*FLA11* N-glycosylation A mutation vector construction
F11-NglyA-Mu_1R	tgcagaagcGAGCTGAGTGTTGATTTGGTCTG	
F11-NglyA-Mu_2Fnew	ATCAACACTCAGCTCgcttctgcaTCGAGTAATGGCTTAACCGTGT	
F11-NglyA-Mu_2R	tgcgatcgcAACTTGGTTACCGGAGCTagcgatggcAAGAGGGAATTTACCGTTTTGG	
F11-NglyA-Mu_3F	gccatcgctAGCTCCGGTAACCAAGTTgcgatcgcaACTGGAGTTGTCAGCGCCAC	
pGreenNotIOCS-R	CTGGAGCTCCACCGCG	
pGreenKpnI35S-F	CGACTCACTATAGGGCGAATTG	*FLA11* N-glycosylation B mutation vector construction
F11-NglcB-Mu_1R	ttgGAGCTGAGTGTTGATTTGGTCTG	
F11-NglcB-Mu_2Fnew	ACCAAATCAACACTCAGCTCcaaTCTTCCTCGAGTAATGGCTTAAC	
F11-NglcB-Mu_2R	ttgAACTTGGTTACCGGAGCTAGTGATctgAAGAGGGAATTTACCGTTTTGG	
F11-NglcB-Mu_3F	cagATCACTAGCTCCGGTAACCAAGTTcaaATCACCACTGGAGTTGTCAGC	
pGreenNotIOCS-R	CTGGAGCTCCACCGCG	

Supplemental Table S3. List of vectors used for domain swaps.

Vector ID	Description	Purpose
ARV1	pGreen0179-*proFLA11*::spFLA11-His-YFP-FLA3	Promoter swap study
YMV261	pGreen0179-*proFLA11*::spFLA12-His-YFP-FLA12	
YMV171	pGreen0179-*proFLA11*:: spFLA11-His-YFP-FLA11AG1-FAS12-FLA11AG2-FLA11GPI	FAS1 swap study
ARV2	pGreen0179-*proFLA11*::spFLA11-His-YFP-FLA11AG1-FAS3-FLA11AG2-FLA11GPI	
YMV162	pGreen0179-*proFLA11*::spFLA11-His-YFP-FLA11AG1-FAS11-FLA11AG2-FLA11 linker-FLA12GPI	AG2 and GPI swap study
YMV173	pGreen0179-*proFLA11*::spFLA11-His-YFP-FLA11AG1-FAS11-FLA11AG2-FLA12 linker-FLA12GPI	
YMV172	pGreen0179-*proFLA11*::spFLA11-His-YFP-FLA11AG1-FAS11-FLA12AG2-FLA12 linker-FLA12GPI	
ARV3	pGreen0179-*proFLA11*::spFLA11-His-YFP-FLA11AG1-FAS11-FLA3AG-FLA3GPI	

Supplemental Table S4. List of primers used for domain swap vector constructs.

Name	Primer sequence	Purpose
ARP9	GCGGAGGTGGGTCACCTAGGGTTAACATAACCCGAGTGCTCG	ARV1
ARP2	ATATCTCATTAAAGCAGGACtctagaTTAGAACCCAACAAAGCTAGCCA	
ARP10 Nos	GTCCTGCTTTAATGAGATATGC	
pGreen-OCS-R	CTGGAGCTCCACCGCG	
YMV171-FAS12-F	CAGGCGGAGGTGGGTCAcctaggCAGGCTCCAGCTCCAGGCCCTTCAGGTCCCACAAACGTTACCAAAATCCTAGAG	YMV171
YMV171-FAS12-R	CAAAACCTTATCGACCTGATAAACAGCG	
YMV171-F11AG-F	ATCAGGTCGATAAGGTTTTGCTGCCATTAGCCATGTTTGGATCAAG	
pGreen-OCS-R	CTGGAGCTCCACCGCG	
ARP3	CGGTTCAGGCGGAGGTGGGTCAcctaggCAGGCTCCAGCTCCAGGCCCTTCAGGTGTTAACATAACCCGAGTGCTCG	ARV2
ARP4	TTTCACACCAGAACCAAAGTAAATC	
ARP5	GATTTACTTTGGTTCTGGTGTGAAACTGCCATTAGCCATGTTTGG	
pGreen-OCS-R	CTGGAGCTCCACCGCG	
YMV111proF11-F	ACTATAGGGCGAATTGGGTACCcagcagcgtagatcttttgagtg	YMV162
162F11-R	ACGCAAAACGACACCGCATCACACACAGAACCAACATTACGCAAAGCAAACGAAGCATCTGAATCAGTAGAATCTCCTCCATCAT	
162F12GPI-F	GATGCGGTGTCGTTTTGCGTCATGAGTGTAATGCTCGCATGGTTTTATTTGTGATCTAGAGTCCTGCTTTAATGAGAT	
pGreen-OCS-R	CTGGAGCTCCACCGCG	
YMV101-F11-F	CAGGCGGAGGTGGGTCAcctaggCAGGCTCCAGCTCCAGGC	YMV173
YMV173-FAS11AG2_R	AGGGGCCGGAGCAGG	
YMV173-F12linkGPI-F	GTGGCTCCTGCTCCGGCCCCTGTATCGAAATCAAAGAAGAAGAAGGATGACAGT	
pGreen-OCS-R	CTGGAGCTCCACCGCG	
YMV101-F11_F	CAGGCGGAGGTGGGTCAcctaggCAGGCTCCAGCTCCAGGC	YMV172
YMV273-FAS11-R	CAAAACTTGATCAACCTGATAAACGGCC	
YMV273-F12AG-F	ATCAGGTTGATCAAGTTTTGCTTCCACAACAAGTTTTCGATCCTCG	
pGreen-OCS-R	CTGGAGCTCCACCGCG	
ARP7	TCAGCGGTTTGAGGAGCGCCCAAAACTTGATCAACCTGATAAACG	ARV3
ARP8	GGCGCTCCTCAAACCGC	
ARP9	GCGGAGGTGGGTCACCTAGGGTTAACATAACCCGAGTGCTCG	
pGreen-OCS-R	CTGGAGCTCCACCGCG	

Supplemental Table S5. List of primers used for RT-qPCR.

Target gene	Forward primer	Tm (ºC)	Reverse Primer	Tm (ºC)
FLA11 (AT5G03170)	AAGCACTCAAGCCTCAGACC	67	TGAAGGCGTTATCAGTCGGG	67
ACT2 (AT3G18780)	ACATTGTGCTCAGTGGTGGA	67	GAGATCCACATCTGCTGGAAT	64
